# Growth dynamics and complexity of national economies in the global trade network

**DOI:** 10.1038/s41598-018-33659-6

**Published:** 2018-10-15

**Authors:** Gianluca Teza, Michele Caraglio, Attilio L. Stella

**Affiliations:** 1 Dipartimento di Fisica e Astronomia Università di Padova, Via Marzolo 8, I-35131 Padova, Italy; 2 KU Leuven, Institute for Theoretical Physics, Celestijnenlaan 200D, 3001 Leuven, Belgium; 3 Sezione INFN Università di Padova, Via Marzolo 8, I-35131 Padova, Italy

## Abstract

We explore the quantitative nexus among economic growth of a country, diversity and specialization of its productions, and evolution in time of its basket of exports. To this purpose we set up a dynamic model and construct economic complexity measures based on panel data concerning up to 1238 exports of 223 countries for 21 years. Key statistical features pertaining to the distribution of resources in the different exports of each country reveal essential in both cases. The parameters entering the evolution model, combined with counterfactual analyses of synthetic simulations, give novel insight into cooperative effects among different productions and prospects of growth of each economy. The complexity features emerging from the analysis of dynamics are usefully compared with gross domestic product per capita (GDP_pc_) and with an original measure of the efficiency of the economic systems. This measure, whose construction starts from an estimate of bare diversity in terms of Shannon’s entropy function, is made fully consistent with the degree of specialization of the products. Comparisons of this measure with the model parameters allow clear distinctions, from multiple perspectives, among developed, emerging, underdeveloped and risky economies.

## Introduction

A fundamental problem in the study of economic growth is the quantitative assessment of the effect that the variety and quality of goods produced by a country has on its overall productivity^1,2^. This assessment faces the extra difficulty that the productivity depends also on nontradable capabilities and on intangible assets. An advance in this field was recently made within the approach to economic complexity. This consisted in the proposal of a measure of diversity in the productions of a country which takes into account their degree of specialization, as deducible from a comparative analysis at global level^3^. An idea at the basis of this approach^4–7^ is that the productive basket of all countries, if properly analysed, should supply also most of the information encoded in assets like education, quality of life, technological sophistication, or institutions.

In this work we push this view further. Indeed, we consider the data concerning yearly exports of all countries, retrieved from the global trade network, as a complete set of coordinates suitable for a closed description of their evolution in time. As we show below, supplying a dynamic model of all these data allows to make contact with aspects of the economic complexity of the nations, which seem to be hardly revealable by other approaches. At the same time, comparisons of the parameters quantifying these aspects in the dynamic description, can be made with a novel measure of economic complexity extracted directly and reliably from the data whose evolution is described by the model.

Inspired by the success of relatively more simple descriptions used in various fields, like population and evolutionary dynamics^8^, portfolio strategies^9^ or interface growth^10^, we formulate stochastic differential equations for the evolution of the exports of the various countries. The possibility of synthetic simulations opens the way to counterfactual or path dependence analyses. For example, the equations generate through noise effects alternances of periods of favourable and unfavourable conditions for each export, and the occurrence of contextual transfers of resources between different exports, can lead to the result of enhancing or depressing average growth. This mechanism gives rise to explore-exploit tradeoff alternatives in portfolio optimization^9^. Since the model includes a coupling parameter which tunes simultaneously all the rates of resource transfers between different exports, we can estimate as a function of this coupling the potential average growth which could have occurred in a past period, and compare it with the historical one. This is illustrated in the Results Section below for the case of the USA, China and Russia. The counterfactual analysis relative to the period 1995–2015 shows in each case how pronounced are the maxima of average growth and how far they fall from the historically calibrated values of the transfer coupling. This provides an indication of the growth potential of the countries associated with variations of the average transfer rate.

**Figure 1:**
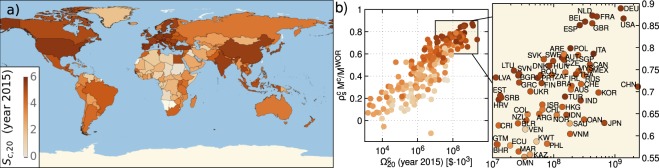
Panel (a) 223 countries colored with a palette related to the entropic measure *S*_*c*_, computed for 2015. Few countries with no data are white. Panel (b) This scatter plot reveals that the total export ${{\rm{\Omega }}}_{20}^{c}$ and the normalized Spearman’s correlator ${\rho }_{s}^{c}{M}^{c}/{M}^{WOR}$ discussed in the text are strictly correlated. The ranking of most advanced countries is close to that valid for globally aggregated exports. The color scheme is the same as the one used in the Panel (a) and shows that *S*_*c*_ is clearly increasing with increasing Spearman’s correlator. In the SI we provide an enlarged figure that shows the country name associated with every point in the plot.

A model similar to that presented here was recently applied to the simpler problem of the time evolution of exports aggregated among all countries at global level^11^. The similarity is due to the circumstance that, at both aggregate and single developed nation level, data show a progressive enforcement and stabilization of a peculiar way in which resources are distributed in different productions. This tendency towards a similar relative distribution of resources in the baskets should be regarded as a main effect of the reciprocal influences of different national economies in the global market. As shown in Fig. 1(b), especially for the most developed countries, the rankings associated with these distributions appear very close to that valid after global aggregation. These distributions in fact constitute a basic ingredient for the construction of our dynamics.

The precise distributions of resources into different exports are not taken into account by approaches which attribute to each country the role of exporter of a given product simply on the basis of sharp threshold criteria. Such criteria have been extensively adopted recently in the construction of economic complexity measures^3–7^. The structure of our dynamical model of export growth suggests to look for complexity measures which fully take into account the precise relative weights of the different exports in the national baskets. In development economics measures of diversity of productions using these weights as arguments of Shannon entropy functions are already known^12,13^. A key result we provide here is the construction of an iterative, safely and rapidly convergent scheme for a consistent simultaneous evaluation of the diversification of national exports and the specialization of products. The starting inputs in the iterative scheme are Shannon entropy indicators of bare diversity and specialization. An achievement in this context is anticipated in Fig. 1(a), where we report in a world map our estimated entropy measure of economic complexity for 223 countries referring to historical data of 2015. The analysis of the various parameters and indexes of our model is considerably helped by comparisons with this novel entropic measure and GDP_pc_ data.

## Distributions of Exports from Individual Countries

For each country and each product we consider the yearly exports realized from 1995 to 2015 (see SI). Limiting here examination to 131 countries whose export data do not show too many interruptions in the whole period, we call ${Z}_{p,n}^{c}$ the total value (in thousands of US-dollars) of the product category *p* (*p* = 1, 2, …, *M*^*c*^) exported in the year *n* (*n* = 0, 1, …, *T* with *T* = 20) by country *c* (*c* = 1, 2, …, *N* = 131). The number of exported products, *M*^*c*^, varies from country to country, but for the most developed economies ${M}^{c}\lesssim 1238\equiv {M}^{WOR}$. An interesting result that emerged in ref.^11^ is that, the exports aggregated at global level, ${Z}_{p,n}^{WOR}={\sum }_{c}\,{Z}_{p,n}^{c}$, are sorted in value according to a ranking which is maintained and slowly stabilizes, up to slight fluctuations, in the years. Its existence and the fact that similar stable rankings are approached by the exports of each individual country, especially the most developed, is a key feature of the organization of economies. For each country, we decide to assign a reference ranking of product *p* based on the fraction of the value of product *p* over the whole country export, ${{\rm{\Omega }}}_{n}^{c}\equiv {\sum }_{p=1}^{{M}^{c}}\,{Z}_{p,n}^{c}$, averaged over the last 5 years:

1 $${z}_{p}^{c}\equiv \frac{1}{5}\sum _{n=16}^{20}\,\frac{{Z}_{p,n}^{c}}{{{\rm{\Omega }}}_{n}^{c}}$$

In order to compare the ranking of the ${z}_{p}^{c}$ of country *c* to that of the exports aggregated at global level in year *n*, ${z}_{p,n}^{WOR}$, we evaluate the Spearman’s rank correlation coefficient^14^ ${\rho }_{s}^{c}$ between the former and the latter set deprived of the goods that are not exported by country *c*. Since an extensive mismatch between the two sets could yield a misleadingly high coefficient, we further multiply the Spearman’s correlation by *M*^*c*^/*M*^*WOR*^. Figure 1(b) reports this quantity coupled with the total export ${{\rm{\Omega }}}_{n}^{c}$ for *n* = 20 (year 2015). It shows that, indeed, developed countries share a common ranking structure, close to that of globally aggregated exports (${\rho }_{s}^{c} \sim 1$). Moreover, with lower total export the correlator decreases.

**Figure 2:**
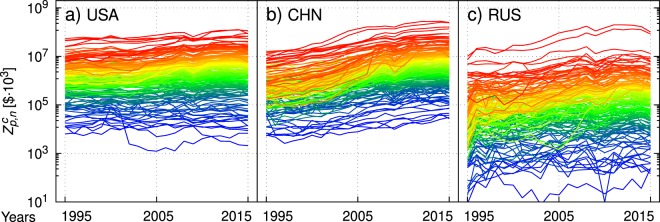
Interpolations of yearly values of products exported by the USA (**a**) China (**b**) and Russia (**c**) from 1995 to 2015. The wavelength of the color for each group of products is proportional to the corresponding ${z}_{p}^{c}$. The relative stability of products rankings in value over time causes marked rainbow effects in all images. For better visualization, the data have been further coarse-grained to roughly 200 categories of products, corresponding to 3 digits of the HS codifications (see SI Appendix).

In Fig. 2, we show the time series of the products exported by the USA (a), China (b) and Russia (c). The wavelengths of the colors used for each product *p* are proportional to the corresponding ${z}_{p}^{c}$: the rainbow image perceived in the figures confirms the existence and stability of the ranking of product values. Exceptions are of course present, corresponding to goods changing their rank in the period. Furthermore, the rainbow effect in the graph is slightly more pronounced for the USA compared to China. This is a sign of the fact that China’s growing economy is still reorganizing its internal structure. Russia instead shows a wider spread of the product export values, indicating that in its case there is a certain number of products whose impact on the country’s economy is low and a relatively smaller number of products with a high impact, like coal, petroleum, natural gas and minerals. Figure 2 also shows that China’s exports increased much faster than those of the USA. In fact, defining the average growth rate over time *T* for a country *c* as

2 $${\lambda }_{T}^{c}\equiv \frac{1}{{M}^{c}\cdot T}\sum _{p=1}^{{M}^{c}}\,\mathrm{log}\,[\frac{{Z}_{p,T}^{c}}{{Z}_{p,0}^{c}}]$$

one gets ${\lambda }_{T}^{USA}=2.1$% and ${\lambda }_{T}^{CHN}=11.2$%. Russia experienced average export growth comparable to China ${\lambda }_{T}^{RUS}=10.5$%.

## Model of Export Dynamics for Individual Countries

Since yearly export records result from variations in much shorter periods, we set up equations in continuous time *t* measured in year units (*t* = 0 corresponds to the end of 1995): the value of product *p* exported by country *c* in the year preceding time *t* is indicated with ${Z}_{p}^{c}(t)$, with 0 ≤ *t* ≤ *T*, (*T* corresponds to 2015) Thus, ${Z}_{p,n}^{c}$ gives a discrete representation of this function of time in 21 points.

We write a stochastic system of equations for the ${Z}_{p}^{c}(t)$’s in the following form.

3 $${\partial }_{t}{Z}_{p}^{c}(t)=[{\eta }_{p}^{c}(t)+{\mu }^{c}(t)]\,{Z}_{p}^{c}(t)+\sum _{p^{\prime} \ne p}[{J}_{pp^{\prime} }^{c}{Z}_{p^{\prime} }^{c}(t)-{J}_{p^{\prime} p}^{c}{Z}_{p}^{c}(t)]$$

The first term in ${Z}_{p}^{c}(t)$ on the rhs of Eq. 3 would enter, in somewhat simplified form, in a geometric Brownian motion description of asset dynamics familiar from the standard model of finance^9,15^. *μ*^*c*^(*t*) represents a deterministic drift, accounting for the average growth of the exports of a given country in the absence of mutual influences. The time-dependence comes from the need to include inflationary effects. Indeed, as explained in the SI, the exports values of a certain year are expressed in the current currency of that specific year. Therefore inflation has to be included as a drift term in the equation. So, we write ${\mu }^{c}(t)={\bar{\mu }}^{c}+I(t)$ in order to separate the constant average contribution to the drift ${\bar{\mu }}^{c}$, from the inflationary one, *I*(*t*). Since all exports in our dataset are expressed in US-dollar, we simply assumed *I*(*t*) to be the same yearly step-wise function for all countries and we take its values from the OECD^16^ (see SI). The multiplicative noise ${\eta }_{p}^{c}$, representing the variability of conditions for the export of product *p*, is correlated in time and between products according to

4 $$\langle {\eta }_{p}^{c}({t}_{1})\,{\eta }_{p^{\prime} }^{c}({t}_{2})\rangle ={c}_{pp^{\prime} }^{c}\,\frac{{({\sigma }^{c})}^{2}}{{\tau }^{c}}\,{{\rm{e}}}^{-|{t}_{1}-{t}_{2}|/{\tau }^{c}}$$

where *τ*^*c*^ represents the typical duration of opportunity/crisis periods^17^ and ${c}_{pp^{\prime} }^{c}$ are the elements of a matrix constructed from the correlations between variations at equal times of the different exports in the database^11^ (see SI).

The second term on the rhs of Eq. 3 represents the mechanisms through which different exports influence each other within a country. This effect, specified by the rates of transfer ${J}_{pp^{\prime} }^{c}$, can be ascribed to various mechanisms such as dependencies between exports due to the organization of the production, investment policies and internal regulations. Once coupled with the correlated noise, this term is responsible for part of the average export growth as measured by Eq. 2. The coefficients ${J}_{pp^{\prime} }^{c}$ have a structure which largely reflects the prevailing distribution of the exports in each country. At the same time, they take into account how strongly the variations of the export values are correlated during the recorded history. Thus, we put ${J}_{pp^{\prime} }^{c}={G}^{c}{z}_{p}^{c}|{c}_{pp^{\prime} }^{c}|$, where *G*^*c*^ is a coupling constant that regulates the magnitude of these transfer processes, and ${c}_{pp^{\prime} }^{c}$ is the same matrix entering in Eq. 4. This structure makes the transfer term consistent with Tinbergen gravity law^11^ (see SI). The proportionality of ${J}_{pp^{\prime} }^{c}$ to ${z}_{p}^{c}$ guarantees that the solutions, in absence of noise, tend to a long time attractor with relative weights in the distribution precisely proportional to the ${z}_{p}^{c}$’s (see SI). So far, the model is described by a set of 4 parameters, which characterize in detail different properties of a country’s economy. *G*^*c*^ is an indicator of the average rate of transfers of resources occurring between different classes of products. ${\bar{\mu }}^{c}$ takes into account the average yearly net input of resources (natural, labor, financial, etc.) devoted to the production of exports. Finally, *σ*^*c*^ and *τ*^*c*^ quantify, respectively, the amplitude and the typical correlation time of the fluctuations faced by different products. The values of these parameters are determined by best fits of the historical records. We notice that all the parameters to be fitted in the model, and rates ${J}_{pp^{\prime} }^{c}$, are assumed to be time independent, in a sort of mean field spirit. This is justified by the considerable complexity of the problem. Introducing a time-dependence for the ${J}_{pp^{\prime} }^{c}$ in particular, would in principle allow to explore optimization strategies of the average growth more realistic than those one can test upon tuning only the constant value of *G*^*c*^ for the whole period, as discussed in the next Section. Indication that such dependence on time could be a realistic feature of a more sophisticated model is provided by the often large variances displayed by the yearly records whose average yields our matrix ${c}_{pp^{\prime} }^{c}$. In spite of this, we take these averages as time-independent matrix elements, in a spirit which is not far from that of a recent study of the multi-layered network structure underlying financial and macroeconomic dynamics^18,19^. When dealing with the data of certain countries we find that the calibration of these parameters reveals the presence of a noise that cannot be fully reproduced by the dynamics of Eq. 3 (see SI for more details). This occurs especially in the case of less developed countries since when a country is not driving in the global economy, the fluctuations of its productions can be strongly affected by the dynamics of other countries, an effect not explicitly included in our model. For this reason, in order to properly calibrate the fluctuation parameters *σ*^*c*^ and *τ*^*c*^, we need to clean the data by removing these external-noise effects. This suggests us to introduce an additional, fifth parameter, ${\sigma }_{0}^{c}$, which in turn becomes an interesting indicator of the role of a country in the World economy scene, by quantifying its sensibility to other countries’ influence.

## Results

### Growth

Concerning the average growth introduced in Eq. 2: one would like to determine the separate contributions of the terms in Eq. 3. To this purpose one can divide Eq. 3 by ${Z}_{p}^{c}(t)$ and integrate it in the interval [0, *T*] and take the average over the set of products. This allows to obtain:

5 $${\lambda }_{T}^{c}={h}_{T}^{c}({G}^{c})+{\bar{\mu }}^{c}+\frac{1}{T}{\int }_{0}^{T}\,I(t)dt$$

where ${h}_{T}^{c}$ results from integration of the transfer terms. This term depends strongly on *G*^*c*^ and can be estimated by discrete summations (see SI). Thus, as anticipated, part of the growth is directly associated with the cooperative transfer terms in the equations. Of course, the effect of transfers on growth depends also on the fluctuations caused by the colored noise. The combination of the two factors may realize conditions in which the positive trends of some productions are optimally exploited to increase the growth. So, besides determining *G*^*c*^ and ${h}_{T}^{c}({G}_{c})$ on the basis of the historical data, it makes sense to simulate histories in which, e.g., *G*_*c*_ is varied, while keeping historical initial conditions and other parameters fixed at the calibrated values. Thus, one can deduce the effect that a variation of *G*^*c*^ could have had on the average growth. This is interesting and not devoid of prescriptive value, since *G*_*c*_ can be in principle partly controlled, e.g., by regulations and investment policies.

**Figure 3:**
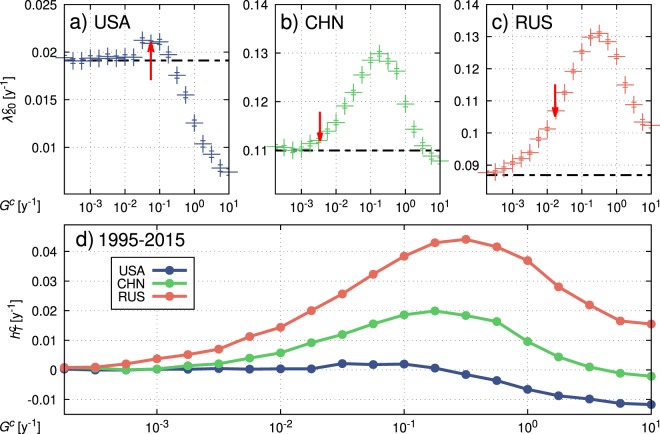
Panels (a,b and c) report the average growth ${\lambda }_{20}^{c}(G)$ of the USA, China and Russia, respectively. They are obtained with simulations for different values of the transfer parameters *G*^*c*^. Red arrows point to the calibrated values of *G*^*c*^ and the growth estimated from historical data. The black dashed lines represent the deterministic drift contributions to the average growth. ${\bar{\mu }}^{c}+\frac{1}{T}{\int }_{0}^{T}I(t)t$. Panel (d) shows the previous curves deprived of the drift, in order to confront the growth contributes ${h}_{20}^{c}$ of cooperative nature.

In Fig. 3, we report such plots for ${\lambda }_{20}^{c}$ of the USA (a), China (b) and Russia (c) indicating also the values of *G*^*c*^, (abscissa pointed by the red arrow in panels a,b,c), of ${\lambda }_{20}^{c}$, (y-coordinate of red arrow tip), and of ${\bar{\mu }}^{c}$ plus average inflation rate (horizontal black dashed line) as determined by calibration on historical data. The agreement of the arrow tips with simulation data points confirm the consistency of the calibrated model dynamics. The distance between the level of the black dashed line and the data points give a measure of the contribution to the growth to be ascribed to the transfer terms in the equations. This contribution appears positive in the historical cases, but becomes always negative for sufficiently high *G*^*c*^ values. Remarkably, for the USA the contribution of the transfer term is very small, and not susceptible of sensible increments upon an increase of *G*^*c*^. The situation is very different for China and Russia, where we recognize a much larger contribution of the transfers and, most important, a substantial increment of the growth with larger *G*^*c*^’s. Figure 3(d) reports the plots for the three countries after subtraction of the average deterministic contribution.

### Entropic measures

In order to further clarify the meaning of the model parameters we define a quantity with the same scope as that of the Economic Complexity Index (ECI)^4^ or Fitness^5,20^. To be directly comparable with intensive indicators, like GDP per capita, it should be a function of the relative weights of the exports in the distribution of each basket, expressed as ${s}_{c,p}\equiv {Z}_{p}^{c}/{{\rm{\Omega }}}^{c}$, for product *p* and country *c* (for simplicity we drop the year index *n* in this section). The Shannon’s entropy function already in use in development economics as a variety or diversity indicator is a natural candidate^12,13^. This indicator increases with both the number and the evenness of products shares. It has the advantage of being independent of the detailed structure and number of intermediate stages leading to the final repartition of resources^21^, and thus is quite robust. So, as a first step we define a country’s production diversity as ${S}_{c}^{\mathrm{(0)}}=-\,{\sum }_{p}\,{s}_{c,p}\,\mathrm{log}\,{{s}}_{c,p}$. Within this analysis we can include all the countries in the database regardless of time interruptions of the export data, so *N* = 223. An entropic indicator taking into account the specialization of the exports can also be defined as ${Q}_{p}^{\mathrm{(0)}}=\,\mathrm{log}\,N+{\sum }_{c}\,{q}_{c,p}\,\mathrm{log}\,{q}_{c,p}$, where ${q}_{c,p}\equiv {Z}_{p}^{c}/{Z}_{p}^{WOR}$ are the shares of country *c* evaluated with respect to the overall global export of product *p*. Here we exploit the fact that the maximum Shannon entropy of a product is log (*N*). Thus, ${Q}_{p}^{\mathrm{(0)}}$ increases with the entropy of the product being more distant from this maximum. As such, it weigths the degree of specialization of product *p*. So far ${S}_{c}^{\mathrm{(0)}}$ depends only on the shares in value of the products produced by the country, and not on their specialization. Analogously, ${Q}_{p}^{\mathrm{(0)}}$ does not depend on how developed are the various countries exporting product *p*. We can introduce such dependences by reweighting the shares entering each of the two quantities through factors depending on the other. So, for example in the case of the product diversity the idea is to start weighting each product *p* by ${Z}_{p}^{c}$ multiplied by a quality factor ${Q}_{p}^{\mathrm{(0)}}$, and to modify consistently the normalization Ω^*c*^. In this way each product enters in the entropy function with the importance deriving from its specialization at global level. For consistency such reweighting should be iterated until a fixed point is reached. The iteration step from stage *k* − 1 to *k* reads:

6 $$\{\begin{array}{rcl}{S}_{c}^{(k)} & = & -\sum _{p}\,\frac{{Z}_{p}^{c}{Q}_{p}^{(k-\mathrm{1)}}}{\sum _{p^{\prime} }\,{Z}_{p^{\prime} }^{c}{Q}_{p^{\prime} }^{(k-\mathrm{1)}}}\,\mathrm{log}\,[\frac{{Z}_{p}^{c}{Q}_{p}^{(k-\mathrm{1)}}}{\sum _{p^{\prime} }\,{Z}_{p^{\prime} }^{c}{Q}_{p^{\prime} }^{(k-\mathrm{1)}}}]\\ {Q}_{p}^{(k)} & = & \mathrm{log}\,(N)+\sum _{c}\,\frac{{Z}_{p}^{c}{S}_{c}^{(k-\mathrm{1)}}}{\sum _{c^{\prime} }\,{Z}_{p}^{c^{\prime} }{S}_{c^{\prime} }^{(k-\mathrm{1)}}}\,\mathrm{log}\,[\frac{{Z}_{p}^{c}{S}_{c}^{(k-\mathrm{1)}}}{\sum _{c^{\prime} }\,{Z}_{p}^{c^{\prime} }{S}_{c^{\prime} }^{(k-\mathrm{1)}}}]\end{array}$$

The algorithm has a unique fixed point, *S*_*c*_ and *Q*_*p*_ to which both ${S}_{c}^{(k)}$ and ${Q}_{p}^{(k)}$ converge rapidly, as we tested numerically (see SI). This fixed point yields our consistent entropic measures of productions diversity and products specialization. In Fig. 1b the colors of points representing the various countries give a measure of how large *S*_*c*_ is. We find high values for the most developed countries at the upper right corner of the box. In general, moving vertically in the box one finds a gradient of *S*_*c*_, which should be expected because the correlation with the ranking of globally aggregated exports increases. Significant is also the gradient for horizontal moves. However, some countries to the right, with a larger total export, have in this case lower *S*_*c*_ than those to the left, because their exports are less balanced, and dominated, e.g., by petroleum (like Saudi Arabia, Venezuela, Kuwait etc…). A comparison between our entropic measure and the Fitness *F*_*c*_ defined in ref.^5^ is reported in the SI, where we show that a relation ${F}_{c}={e}^{{\rm{const}}\cdot {S}_{c}}$ is very plausible (the Spearman correlation index between *F*_*c*_ and *S*_*c*_ has the value ${\rho }_{s}\simeq \mathrm{95 \% }$).

Our definitions of *S*_*c*_ and *Q*_*p*_ differ from those of the analogous quantities constructed within the economic complexity approach in a basic aspect: while in these approaches the quantities are defined directly as weighted averages, in our case weights are used to renormalize arguments to insert in indicators related to the Shannon entropy function. The use of this function introduces an essential ingredient of non-linearity^5^ in the iterations and guarantees at the same time global stability and smoothness of *S*_*c*_ and *Q*_*p*_. Thus, it provides also a way out of some mathematical problems which occasionally affect the previous algorithms^22^. *S*_*c*_ can also be straightforwardly evaluated at different levels of fine graining of the export data (number of digits used for coding the products), allowing consistent quantification of inter- and intra-sectoral contributions to diversification^13^. Finally, another advantage of our entropic measures is the fact that they make use of the full information contained in the ${Z}_{p}^{c}$’s values, without resorting to binarizations through threshold criteria based on Revealed Comparative Advantage RCA^23,24^.

### Calibrated model parameters

**Figure 4:**
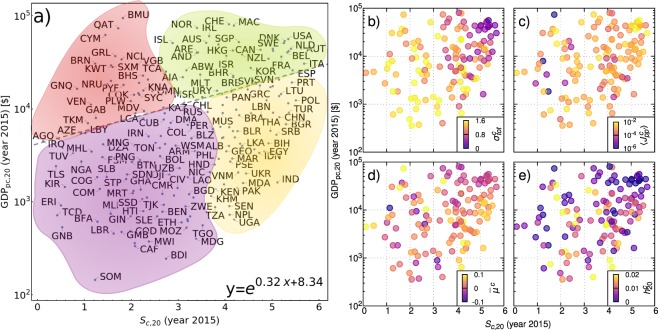
Panel (a) *S*_*c*_ and GDPpc for 2015 allow to distinguish among developed (green), emerging (yellow), underdeveloped (purple) and economically risky (red) countries. In panel (b–e) the colors used are representative, respectively, of: the overall (inner and outer) fluctuations of a country ${\sigma }_{tot}^{c}$, the average transfer $\langle {J}_{pp^{\prime} }^{c}\rangle$, the drift ${\bar{\mu }}^{c}$ and the cooperative growth term ${h}_{20}^{c}$. The parameters, together with the entropic complexity, help us in assessing the correct level of development of a country. In the SI we provide enlarged figures whic show the country name associated with every point in the plot.

Entropic complexity and GDPpc provide an obvious benchmark plane to assess levels of development and stability of countries, even if, of course, one could consider also other quantities, like the eigenvector centrality determined recently in the network study of refs^18,19^. Figure 4(a) reports positions on this plane of 223 countries in 2015. An exponential fit (grey dotted line) shows that GDPpc increases on average with *S*_*c*_, although a precise relation does not hold. The four colored regions are consistent with the model parameters comparisons reported in Fig. 4(b–e). The boundaries are drawn based on the exponential fit of *y* vs *x* indicated in the figure (horizontal), and on a reproduction in our $({S}_{c},{{\rm{GDP}}}_{{\rm{pc}}})$ plane of a similar plot in the plane $({F}_{c},{{\rm{GDP}}}_{{\rm{pc}}})$ of ref.^20^. In Fig. 4(b) the 131 representative points of the calibrated countries are colored from a palette defined by the values of the total noise amplitude, ${\sigma }_{tot}^{c}=\sqrt{{({\sigma }^{c})}^{2}{\tau }^{c}+{({\sigma }_{0}^{c})}^{2}}$. The noise strength is rather low in the upper right corner (developed countries), and very large in the lower left one (risky economies). The vertical gradient is present also on the right side, where, however, some developing countries, like China and India, show relatively low noise effects. Panel (c) reports the average annual transfer rate of resources between exports, $\langle {J}_{pp^{\prime} }^{c}\rangle ={\sum }_{pp^{\prime} }{J}_{pp^{\prime} }^{c}/{M}^{c}$, an indicator of the flexibility of the economy. This is always large for developed countries. One finds exceptionally high values also for some less developed countries. Panel (d) reports ${\bar{\mu }}^{c}$, the average annual rate of resources input. This is only one of the contributions to the slope seen, e.g., in the time series in Fig. 2. It is rather low for countries in the upper right corner. This should not surprise, since ${\bar{\mu }}^{c}$ is not a usual growth indicator, like GDPpc, but rather quantifies the average rate of investment of resources for the growth net of inflation and cooperativity effects. It is much higher in the lower right corner, where emerging economies with high *S*_*c*_ are located. On the left vertical side countries with low entropic complexity have a mixed behavior, signaling that these are dynamically far from stationarity. The low value of ${\bar{\mu }}^{c}$ for developed economies means that, when the country approaches a state with high entropy and high GDPpc, fresh resources for growth start diminishing. A similar effect has been clearly observed in ref.^11^ for exports aggregated at global level over a period of 39 years. The indicator ${h}_{T}^{c}$ is reported in panel (e), showing that developed economies have a relatively low contribution to growth coming from cooperative effects. This is consistent with the fact that for these countries the margins of optimization of growth upon variation of *G*^*c*^ are rather narrow, as seen, e. g., in the counterfactual analysis for the USA (Fig. 3(a)). In particular ${h}_{T}^{c}$ remains very small compared to the total drift inclusive of inflation. As already remarked above, ${h}_{T}^{c}$ depends on combined effects of the transfers and of noise. Higher values of ${h}_{T}^{c}$ are found for countries with low GDPpc and medium *S*_*c*_. Thus, relatively low transfer rates combined with high levels of noise can produce relevant cooperative growth effects.

## Discussion

The data driven approach to growth proposed here has features and implications worth of further investigation in both development economics and network theory. Our dynamic model provides novel insight into the complexity of economic systems and into the role played by cooperative effects in growth. The idea that the productive output of a country, if weighted in terms of competitiveness, allows to fully account for endowments^3,5^, has been extended to the dynamic context. Indeed, the basic assumption made here is that, for each country, the export basket structure with its fluctuations should also be sufficient to determine the statistics of its evolution in time. The model embodies explicit interaction effects between productions which are found to be an essential ingredient of growth dynamics, but are generally not addressed in the economic literature. Such effects play a key role in enhancing or depressing the growth due to their interplay with the variability of market conditions. The fact that the long term dynamics is closely controlled by the relative distribution of resources in the export basket of each country suggests that interventions aimed at increasing efficiency of an economic system should stably modify the structure of its basket, making it as similar as possible to those of the most efficient economies. The effects of such modification should be testable by simulations of our model.

Besides inspiring the dynamic model construction, the focus on the distribution of resources in different exports suggested a novel way to obtain measures of complexity based on the full information content of the data. We showed here that a tool to estimate variety familiar in development economics, namely the Shannon entropy function^13^, can be used to evaluate the diversity of productions consistently with the specialization of products. The resulting entropic measures, *S*_*c*_ and *Q*_*p*_, do not rely on RCA threshold criteria^23,24^ and are nicely convergent and stable. In the broader context of the theory of networks, the use made here of the Shannon entropy function to extract these measures is fully original with respect to previous applications aimed at characterizing topological heterogeneity^25^, or at assessing the statistical significance of monopartite projections of bipartite networks^26^. Our results open a novel, entropy based way to explore the properties of bipartite networks so frequently met in the real world^27^.

Our entropic measures are derived directly from those distributions identified here as the main generators of dynamics. This strongly supports the expectation that these measures should embody essential information concerning economic growth and should be good candidates as appropriate collective variables for describing this phenomenon in low dimensionality spaces^20^. Thus, the nexus explored in this work is deep and promises to further improve our understanding of growth complexity.

## Methods

The data used for quantifying the products exported by each country are extracted from the BACI database^28^, which consists in a revised version of the freely accessible COMTRADE database^29^. Gross Domestic Products were taken directly from a database^30^ redacted by the United Nations Statistics Division (UNSD), while the Consumer Price Index (inflation) data were provided by the Organisation for Economic Co-operation and Development (OECD)^16^. The calibration procedure for the country parameters mainly follows the one presented in^11^, with the only difference being in the presence of the additional noise parameter ${\sigma }_{0}^{c}$. A second order Runge-Kutta scheme in the Itô prescription was chosen as a compromise between accuracy and performance for integrating numerically the SDE of Eq. 3. In the SI we provide more details on the databases and the techniques used.

## Electronic supplementary material

Supplementary Information
