# Supplementary material for: Growth dynamics and complexity of national economies in the global trade network
Source: Sci Rep. 2018 Oct 15;8:15230. doi: 10.1038/s41598-018-33659-6 (PMC6189074; doi:10.1038/s41598-018-33659-6)
Supplement: Supplementary file 1 — Supplementary Information [file 41598_2018_33659_MOESM1_ESM.pdf]

Supplementary information for: Growth dynamics and complexity of national economies in the global trade network

Gianluca Teza[1], **Michele Caraglio**[2], **and Attilio L. Stella**[1,3,*]

[1]Dipartimento di Fisica e Astronomia Università di Padova, Via Marzolo 8, I-35131 Padova, Italy
[2]KU Leuven, Institute for Theoretical Physics, Celestijnenlaan 200D, 3001 Leuven, Belgium
[3]Sezione INFN Università di Padova Via Marzolo 8, I-35131 Padova, Italy
[*]stella@pd.infn.it

Introduction

Hereby we provide information on the sources used to extract the data for each single country. We also illustrate the integration method used for the stochastic differential equations and present details of the calibration procedure followed in order to determine the values of the parameters that best fit the data. Additional results and plots illustrating properties of the entropic measures are reported in the last section.

S1 Original databases

S1.1 Export Data

The data used for quantifying the products exported by each country are extracted from the BACI database[1]. This database, redacted by the CEPII Research Institute, is in turn built upon the COMTRADE database (freely accessible at the UN COMTRADE website[2]), and consists in a revision of the latter, in which the authors check and rectify the amounts declared by the exporting and importing countries to make them consistent with each other. All the prices are expressed in terms of current US dollars (thus, justifying the insertion of an inflation term in the dynamic model). The BACII database is a 4 dimensional panel that spans the following axes:

1. Time: data are reported on an annual basis (starting from 1995) and are constantly updated with the latest data available. At the time we started performing our analysis the database covered a 21 years range, with the latest data being those of 2015.

2. Product: The products are classified according to a 6 digit code (the Harmonized System 2007[3]) which consists in roughly 5000 different categories of products.

3. Exporting country: about 223 countries are present in the database. Data are organized at a country-to-country level, so that one can know how much of a single product has been exported from any country to any another.

4. Same for importing countries.

S1.2 Gross Domestic Product

The values of the Gross Domestic Product Per Capita ($\mathrm{GDP_{pc}}$), have been extracted from a database[4] of the United Nations Statistics Division (UNSD). This database spans the period 1970-2015 and quantities are expressed in current US dollars (same as for the BACII database).

S1.3 Consumer Price Index

Finally the Consumer Price Index (CPI) values were given by the Organisation for Economic Co-operation and Development (OECD)[5]. In table S1 we report the values used in our analysis, which correspond to a weighted aggregate at a global level of all the CPIs of every country present in the OECD databases.

Table S1. Yearly values (in percentage) of the World aggregate CPI.

year	$I(t)$	year	$I(t)$	year	$I(t)$	year	$I(t)$
1996	5.55	2001	3.59	2006	2.63	2011	2.86
1997	4.69	2002	2.71	2007	2.50	2012	2.24
1998	4.11	2003	2.42	2008	3.68	2013	1.61
1999	3.54	2004	2.35	2009	0.51	2014	1.73
2000	3.95	2005	2.60	2010	1.84	2015	0.59

S2 Data aggregation

The Harmonized System classification allows us to organize the data in less specific categories by simply aggregating products sharing the most significant digits. Our analysis was performed over 1238 "macro" categories built with 4 digit data for exports of each country. Another aggregation has been carried out over the importing countries axis, in order to obtain the $Z^c_{p,n}$ representing the total amount of product p exported by country c in the year n. The entropic measures construction (see section S6) was carried out on the resulting dataset. In order to apply our dynamic model we had to perform a further selection in the $Z^c_{p,n}$ panel. With data coarse-grained to a 4 digit level, it may still happen that some entries become zero. In principle this should indicate that a certain country stops suddenly to export a product. However, such occurrence is more likely related to an intrinsic problem of missing information in the original database: the data we work on are aggregated, so, if a product disappears, it means mean that the country has ceased to produce a whole macro category of products in just one year, which is unlikely to happen. Moreover these zeroes appear mainly when dealing with underdeveloped countries. So, this suggests that the problem lies in the redaction of the database itself. Having a $Z^c_{p,n} = 0$ is an issue because it produces a singularity when inserted in Eq. S1. So, when this happens, we need to remove the entire time-series associated with that product. We decided to proceed with the calibration only for those countries who kept more than 80% of their exports after these removals. So, the dynamic model was applied to 131 countries in total.

S3 Dynamic model

We rewrite our stochastic system of differential equations (SDE) for the $Z^c_p(t)$'s in the following form:

$$\begin{aligned}\partial_t Z^c_p(t) &= \sum_{p'\neq p}\left[J^c_{pp'}Z^c_{p'}(t) - J^c_{p'p}Z^c_p(t)\right] + \left[\eta^c_p(t)+\mu^c(t)\right]Z^c_p(t)\\ &= \sum_{p'} A^c_{pp'}Z^c_{p'}(t) + \left[\eta^c_p(t)+\mu^c(t)\right]Z^c_p(t)\end{aligned} \tag{S1}$$

where η^c is a colored zero-mean Gaussian noise. In the following sections we provide an exhaustive description of the dynamics described by this system of equations in order to give a better understanding of the meaning of the parameters and of the model ingredients.

S3.1 Correlation matrix

In the basket of a country, this matrix plays the important role of quantifying the correlations between the increments of each pair of exported products. The first step in building such matrix is to evaluate the yearly logarithmic returns

$$R^c_{p,n} = \log\left[\frac{Z^c_{p,n}}{Z^c_{p,n-1}}\right] \tag{S2}$$

which we standardize referring to averages over all the years (indicated by $\langle A_n\rangle = \Sigma_n A_n/T$ for a quantity A_n).

$$r^c_{p,n} = \frac{R^c_{p,n} - \langle R^c_{p,n}\rangle}{\sqrt{\langle (R^c_{p,n})^2\rangle - \langle R^c_{p,n}\rangle^2}} \tag{S3}$$

The correlation between the products p and p' is thus defined as

$$c^c_{pp'} \equiv \langle r^c_{p,n} r^c_{p',n}\rangle = \frac{1}{T}\sum_{n=1}^{T} r^c_{p,n} r^c_{p',n} \tag{S4}$$

where $T = 20$ represents the last available year for the returns (2015 in this case). As we will see in the following sections, the correlation matrix will play an important role in the construction of both the matrix ruling the transfers between different productions and the colored noise.

S3.2 Matrix $J^c_{pp'}$

The matrix $J^c_{pp'}$ is constructed exploiting the correlation matrix as a sort of "inverse distance" (see Timbergen's gravity law[6]): the shorter this "distance", the larger the rate at which the transfer of resources between two products occurs. In the structure of the transfer term of the equations , the gravity law is present also because the transfer term is proportional to both $Z^c_{p'}$ and z^c_p. Indeed, we write:

$$J^c_{pp'} = G^c z^c_p |c^c_{pp'}| \tag{S5}$$

where G^c is a coupling multiplicative constant that regulates the magnitude of these transfer processes, and z^c_p is the fraction of total export for product p averaged the last five years:

$$z^c_p = \frac{1}{5} \sum_{n=16}^{20} \frac{Z^c_{p,n}}{\sum_{p'} Z^c_{p',n}} \tag{S6}$$

This is the quantity used to discuss the ranking in value of exports in the main text. The transition matrix $\mathbf{A}^c$, with elements $A^c_{pp'} = J^c_{pp'}$ for $p' \neq p$ and $A^c_{pp} \equiv -\sum_{p' \neq p} J^c_{p'p}$, establishes and maintains the observed ranking of the various $Z^c_p(t)$'s once we choose $J^c_{pp'} \propto z^c_p$. Indeed, with such a choice, z^c_p is in the kernel of $\mathbf{A}^c$ and, in the absence of noise terms, the solutions of Eq. S1 tend to a long-time attractor in which the Z^c_p's are in the kernel of $\mathbf{A}^c$ itself[7] and are thus proportional to the z^c_p's.

S3.3 Noise

The noise that defines the stochastic dynamics of Eq. S1 needs to take into account that fluctuations in the growth conditions are correlated both in time and between different products. The former correlations require to define η^c as a colored noise with characteristic time τ^c and variance σ^c, while the latter will imply that we can't use a Kronecker delta for the correlation among noises for different products. Indeed, fluctuations affecting a certain product will be felt by other products that are "spatially" close. For these reasons, we define η^c as a colored Gaussian noise with mean and correlation

$$\langle \eta^c_p(t)) \rangle = 0 \tag{S7}$$

$$\langle \eta^c_p(t_1)\, \eta^c_{p'}(t_2) \rangle = c^c_{pp'} \, \frac{(\sigma^c)^2}{\tau^c} \, \mathrm{e}^{-|t_1 - t_2|/\tau^c} \tag{S8}$$

τ^c represents a typical opportunity/crisis period duration[8], while σ^c regulates the magnitude of the fluctuations. Here the averages indicated by $\langle . \rangle$ have the usual meaning in stochastic differential calculus. $c^c_{pp'}$ appears in the noise correlator because the noise is directly responsible for the return correlations recorded historically.

S3.4 Drift

The term $\mu^c(t)$ represents a deterministic drift, accounting for the average percentual input of resources in the export production in a given country in the absence of mutual influences. The time dependence comes from the inclusion of the inflationary effects to which the exports are exposed every year (as explained in section S1) Thus, we write

$$\mu^c(t) = \bar{\mu}^c + I(t) \tag{S9}$$

in order to separate the constant, average contribution to the drift $\bar{\mu}^c$ from the inflationary one $I(t)$, which can be read as an yearly step-wise function whose values are taken from the OECD[5] and reported in table S1.

S4 Calibration procedure

In this section we will drop the $\cdot^c$ to avoid redundancy in the notations. The calibration procedure we follow is similar to that presented in ref.[9], with the main difference lying in the presence of the new background noise σ_0. Dividing Eq. S1 by $Z_p(t)$ and integrating in the time interval $[n_1, n_2]$ (n_1 and n_2 integers), we obtain

$$f_p(n_1, n_2) = G g_p(n_1, n_2) + \bar{\mu} + \frac{1}{n_2 - n_1} \int_{n_1}^{n_2} \eta_p(t) \, \mathrm{d}t \tag{S10}$$

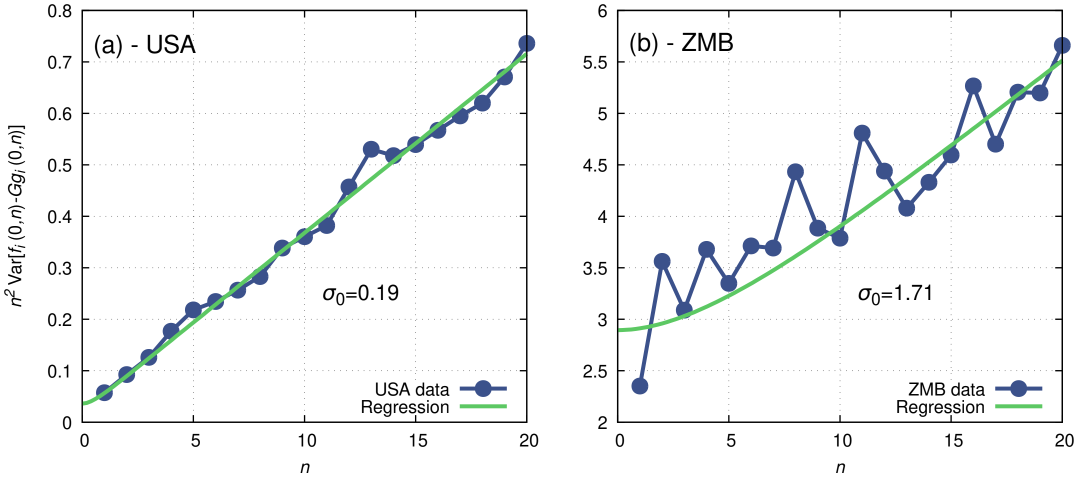

Figure S1. An offset noise term characterize the networks of underdeveloped countries. Here we report the quantity $n^2\text{Var}\left[f_p(0,n) - Gg_p(0,n)\right]$ evaluated from the data of a driving country in the economical scene (USA, panel (a)) and for an underdeveloped country (Zambia, panel (b)). It is evident that an offset term arises for the latter country, justifying the introduction of the additional parameter σ_0.

where we introduced the functions

$$f_p(n_1,n_2) = \frac{1}{n_2 - n_1}\left(\log\frac{Z_{p,n_1}}{Z_{p,n_2}} + \sum_{n=n_1}^{n_2} I_n\right) \tag{S11}$$

$$g_p(n_1,n_2) = \frac{1}{n_2 - n_1}\sum_{\substack{n=n_1\\ p'\neq p}}^{n_2-1}\left[\frac{z_p\left|c_{pp'}\right|}{2}\left(\frac{Z_{p',n}}{Z_{p,n}} + \frac{Z_{p',n+1}}{Z_{p,n+1}}\right) - z_j\left|c_{pp'}\right|\right] \tag{S12}$$

which are estimated approximately by sums in place of integrals because of the discrete nature of the data at our disposal. We immediately see that Eq. S10 establishes a linear relation between such functions, with G representing the slope. Therefore we perform a robust linear regression of the scatter plot f vs. g to estimate the value of G. The intercept obtained with the regression is a first estimate of $\bar{\mu}$ (the stochastic term here averages to 0), but a more accurate derivation can be performed after calibrating the parameters σ and τ. In order to do that, we first need to rearrange Eq. S10 by moving to the left side the Gg_p term. We then evaluate the variance of the resulting equation. By setting $n_2 = n$ and $n_1 = 0$ we obtain:

$$v(n) := n^2\text{Var}\left[f_i(0,n) - Gg_i(0,n)\right] = 2\sigma^2\left(n + \tau\left(e^{-n/\tau} - 1\right)\right) \tag{S13}$$

When dealing with developed countries this relation is usually quite solid, but for the rest of the cases the empirical plots show that there is a missing offset in the equation (see Fig. S1). Such effect is imputable to the fact that the countries are not a closed system and are subject to what happens outside of their borders: the less a country is driving in the global economy, the more its trades will be affected by fluctuations of the countries it relates to. In order to correctly calibrate the parameters σ and τ of a country, we thus need to clean the data by introducing an offset noise term σ_0, which in turn will be an indicator of the extra fluctuations the country's exports are exposed to. Eq. S13 becomes then:

$$v(n) = 2\sigma^2\left(n + \tau\left(e^{-n/\tau} - 1\right)\right) + \sigma_0^2 \tag{S14}$$

The exponential term decays quite fast (ex post analysis shows that $\langle\tau\rangle_c = 2.8 \pm 0.2$ y). So, by neglecting the first years terms, we are able to perform a linear regression: the slope $m = 2\sigma^2$ can be directly inverted to obtain the value of σ, while the intercept equation $q = -2\sigma^2\tau + \sigma_0^2$ needs to be coupled with another condition in order to determine the remaining two parameters. For this reason we integrate Eq. S15 in the full time range $[0,T]$ obtaining:

$$V = \frac{1}{2}\sum_{n=0}^{T-1}\left[v(n) + v(n+1)\right] = 2\sigma^2\left(\frac{T^2}{2} - \tau T + \tau^2\right) + T\sigma_0^2 \tag{S15}$$

where we disregarded the exponential term $e^{-T/\tau}$ obtained after the integration. Inverting the system of equations finally yields

$$\tau = \sqrt{\frac{V - Tq}{2\sigma^2} - \frac{T^2}{2}}\ ,\ \sigma_0^2 = q + 2\sigma^2\tau \tag{S16}$$

We calibrate the last remaining parameter $\bar{\mu}$ through repeated synthetic simulations (see below) of the system of equations deprived of the deterministic drift $\bar{\mu}$ itself. In this case the average growth reads $\lambda_T^* = h_T(G) + \sum_{t=0}^{T} I_t$. Thus, one can find the value of $\bar{\mu}$ that reproduces correctly the growth from the historical data by difference:

$$\bar{\mu} = \lambda_T - \lambda_T^* \tag{S17}$$

In Figs. S5 we report on the global maps the values of the parameters of the 131 countries on which we performed the calibration procedure, while in Figs. S6, S7, S8 and S9 we show a scatter plot on the GDP$_{\text{pc}}$-$S_{c,20}$ plane of the same parameters.

S5 Integration

The stochastic differential Eq. S1 involves a colored Gaussian noise that evolves according to

$$\eta_p(t) = \rho\eta_p(t - \mathrm{d}t) + \sqrt{1-\rho^2}\frac{\sigma}{\sqrt{\tau}}\xi_p(t) \tag{S18}$$

where $\rho \equiv e^{-\mathrm{d}t/\tau}$ and $\xi_p(t)$ is a zero-mean Gaussian noise with correlation $\langle \xi_p(t)\xi_{p'}(t')\rangle = c_{pp'}\delta(t-t')$. In order to obtain a noise with this properties we perform the $\mathbf{C} = \mathbf{LDL}^T$ Cholesky decomposition (see for instance[10]) of the matrix $\mathbf{C} \equiv c_{pp'}$, which can be applied to any symmetric matrix and does not require the evaluation of any square roots in the diagonal terms (which may become a problem when dealing with large matrices whit relatively small entries). Thus, we are able to apply the decomposition to a vector of independent Gaussian noises $\vec{\tilde{\xi}}$ to generate $\vec{\xi} = \mathbf{LD}^{1/2}\vec{\tilde{\xi}}$. By substituting the correlated noise in Eq. S1 and discretizing we obtain

$$\Delta Z_{p,t} = a_p(t, \vec{Z}_t)\,\mathrm{d}t + b_p(t, \vec{Z}_t)\Delta W_{p,t} \tag{S19}$$

where we introduced the Wiener processes $\Delta W_{p,t} = \sqrt{\Delta t}\xi_{p,t}$ and the two coefficients

$$a_p(t, \vec{Z}_t) = \left[\sum_{p' \neq p}\left(J_{pp'}\frac{Z_{p',t}}{Z_{p,t}} - J_{p'p}\right) + \rho\eta_{p,t-\Delta t} + \bar{\mu} + I_t\right] Z_{p,t} \tag{S20}$$

$$b_p(t, \vec{Z}_t) = \sqrt{1-\rho^2}\frac{\sigma}{\sqrt{\tau}}\sqrt{\Delta t}Z_{p,t} \tag{S21}$$

With the assumption $\Delta t \ll \tau$ different integration prescriptions yield the same results: we chose a second order Runge-Kutta scheme for the Itô prescription, suited for systems of equations and characterized by both strong and weak convergence of order 1[11]. If we partition the time interval $[0, T]$ in L sub-intervals each of length Δt, the integration approximation $\{\vec{w}_l\}_{l=1\cdots L}$ estimated over such mesh is given by

$$\begin{cases} \vec{w}_0 = \vec{Z}_0 \\ \vec{w}_{l+1} = \vec{w}_l + \frac{\vec{K}_{1,l} + \vec{K}_{2,l}}{2} \\ \vec{K}_{1,l} = \vec{a}(t_l, \vec{w}_l)\Delta t + \mathbf{B_1}\vec{b}(t_l, \vec{w}_l) \\ \vec{K}_{2,l} = \vec{a}(t_{l+1}, \vec{w}_l + \vec{K}_{1,l})\Delta t + \mathbf{B_2}\vec{b}(t_{l+1}, \vec{w}_l + \vec{K}_{1,l}) \end{cases} \tag{S22}$$

Here $\mathbf{B_{1,2}}$ are two diagonal matrices containing the Wiener processes associated with the noise $\Delta W_{p,n} \equiv \sqrt{\Delta T}(\xi_{p,n+1} - \xi_{p,n})$, which have entries

$$\begin{aligned} (\mathbf{B_1})_{pp'} &= \delta_{pp'}\left(\Delta W_{p,n} - S_{p,n}\sqrt{\Delta t}\right) \\ (\mathbf{B_2})_{pp'} &= \delta_{pp'}\left(\Delta W_{p,n} + S_{p,n}\sqrt{\Delta t}\right) \end{aligned} \tag{S23}$$

where $S_{p,n}$ are random variables that can assume values ± 1 with equal probability $p = 1/2$. In all the numerical integrations performed we set $\Delta t = 0.01$, satisfying the condition $\Delta t \ll \tau$.

S6 Entropic measures of diversity and specialization

S6.1 Algorithm

To simplify notations here we omit the subscript n. Thus, our considerations will refer to data for a generic year. The starting point that led to the construction of the algorithm for the entropies presented in the main text, is the idea to use Shannon's entropy[12] as measure of the diversity in the basket of the exported products of a country[13]. Here the role of probabilities is played by the shares $s_{c,p} \equiv Z_p^c / \sum_{p'} Z_{p'}^c$ of the total export by the country in a certain year. Therefore a first, bare measure of diversity will be:

$$S_c^{(0)} = -\sum_{p=1}^{M} \frac{Z_p^c}{\sum_{p'} Z_{p'}^c} \log \left[\frac{Z_p^c}{\sum_{p'} Z_{p'}^c} \right] \tag{S24}$$

The more a basket is equally partitioned among the products, the higher will be the value of this entropy. The ideal (unrealistic) case is therefore the one in which every product has a share $1/M$, which gives the upper bound limit value $\log M$ to this entropy. On the other hand, when all the exports are concentrated in a restricted niche of products (like in oil-exporting countries) the entropy will be small, with lower bound value 0 corresponding to the case in which only one product is exported. We can define an analogous quantity for the products, in order to understand which products are more common, and therefore easy to produce, and which are more rare. The shares are evaluated in this case over the total global export of each product, as $q_{c,p} \equiv Z_p^c / \sum_{c'} Z_p^{c'}$, Hence we define:

$$Q_p^{(0)} = \log(N) + \sum_{c=1}^{N} \frac{Z_p^c}{\sum_{c'} Z_p^{c'}} \log \left[\frac{Z_p^c}{\sum_{c'} Z_p^{c'}} \right] \tag{S25}$$

so that in this way Q will be higher for the most specialized products and lower for the most common ones. The next step in creating the algorithm is to use S and Q respectively as a weight in valuating more refined entropies: the new weighted shares will therefore be:

$$s'_{c,p} = \frac{Z_p^c Q_p^{(0)}}{\sum_{p'} Z_{p'}^c Q_{p'}^{(0)}} \;,\; q'_{c,p} = \frac{Z_p^c S_c^{(0)}}{\sum_{c'} Z_p^{c'} S_{c'}^{(0)}} \tag{S26}$$

Using these shares as entries, we valuate the new entropic diversities $S_c^{(1)}$ and specializations $Q_p^{(1)}$. We then naturally iterate the procedure to further refine our measures. The algorithm at the k-th steps takes the form:

$$S_c^{(k)} = -\sum_p \frac{Z_p^c Q_p^{(k-1)}}{\sum_{p'} Z_{p'}^c Q_{p'}^{(k-1)}} \log \left[\frac{Z_p^c Q_p^{(k-1)}}{\sum_{p'} Z_{p'}^c Q_{p'}^{(k-1)}} \right] \tag{S27}$$

$$Q_p^{(k)} = \log(N) + \sum_c \frac{Z_p^c S^{(k-1)}}{\sum_{c'} Z_p^{c'} S_{c'}^{(k-1)}} \log \left[\frac{Z_p^c S_c^{(k-1)}}{\sum_{c'} Z_p^{c'} S_{c'}^{(k-1)}} \right] \tag{S28}$$

which is the one presented in the main text.

S6.2 Convergence

In panel (a) of Fig. S2 we show the euclidean distance evaluated between consecutive iterations, which is defined as

$$d\left[\left(S^{(k+1)}, Q^{(k+1)}\right), \left(S^{(k)}, Q^{(k)}\right)\right] = \left(\sum_c (S_c^{(k+1)} - S_c^{(k)})^2 + \sum_p (Q_p^{(k+1)} - Q_p^{(k)})^2\right)^{1/2} \tag{S29}$$

where $S^{(k)}$ and $Q^{(k)}$ are the vectors with components $\{S_c^{(k)}\}_{c=1\ldots N}$ and $\{Q_p^{(k)}\}_{p=1\ldots M}$ respectively. As we can see the distance decreases exponentially fast with a constant rate, until the differences between the iterations go below the double precision limit. In order to understand if the fixed point (S_c, Q_p) is unique (and therefore if the algorithm is globally convergent), we run the iterative algorithm with random initial conditions chosen with a uniform distribution in the range $[0, \log M]$ for the S_c's and $[0, \log N]$ for the Q_p's. Then, for every iteration k we determined the distance from the fixed point and found that the relation

$$d\left[\left(S^{(k)}, Q^{(k)}\right), (S, Q)\right] = e^{\alpha k + \beta} \tag{S30}$$

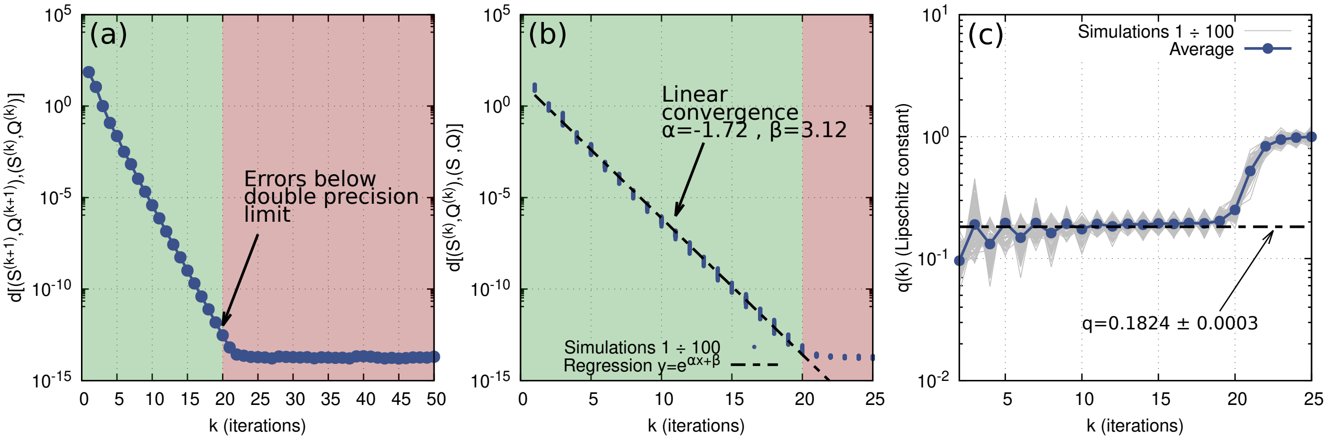

Figure S2. Panel (a): Euclidean distance d evaluated between consecutive steps of the iterative algorithm of the diversities $S_c^{(k)}$ and specializations $Q_p^{(k)}$. The distance decreases exponentially fast until the value $d \sim 10^{-28}$ (around iteration number 20) where the numerical precision of the computer reaches its limit. Panel (b): Distance of iterations from the fixed point of convervence of 100 simulations starting from completely random initial conditions (blue dots). The black dashed line represent the exponential regression $y = e^{\alpha x+\beta}$ in the region where the double precision limit is not exceeded. Panel (c): Valuation of the Lipschitz constant as the ratios of distances from the fixed point of consecutive iterations. The 100 iterations starting from random initial conditions are colored in grey, whereas in blue we see the average performed over such data sample. The dashed line represents the estimation of the Lipschitz constant obtained by the regression illustrated in the previous panel.

holds with coefficients $\alpha = -1.720 \pm 0.001$ and $\beta = 3.12 \pm 0.02$, found by fitting the points obtained with 100 different random initial conditions (see panel (b) of Fig. S2). With this parameters we were therefore able to estimate the Lipschitz constant[14], defined as

$$q \equiv \lim_{k\to\infty} \frac{d\left[\left(S^{(k+1)}, Q^{(k+1)}\right), (S,Q)\right]}{d\left[\left(S^{(k)}, Q^{(k)}\right), (S,Q)\right]} \tag{S31}$$

which turns out to be

$$q = e^{\alpha} = 0.1824 \pm 0.0003 \tag{S32}$$

These estimates suggest to classify the algorithm as globally convergent with a linear rate of convergence ($q < 1$ means that the map associated with the algorithm is a contraction). In panel (c) of Fig. S2 we report the ratios of distances from the fixed points of consecutive iterations, and confront them with the value of the Lipschitz constant q obtained with the previous regression.

S6.3 Data presentation

In Fig. S3 we compare our indicators with the current state of the art in terms of complexity indicators, represented by the fitness F introduced in[15]. As we can clearly see from panels (a) and (b), we have an exceptionally high correlation between F and S: evaluating the Spearman correlator yearly we obtain on average a value of $\langle \rho_s(S,F) \rangle_t = 0.95$, suggesting the existence of a monotonic relation between the two quantities. Such a strong correlation was to be expected, since the algorithm of Fitness has the main purpose of rewarding countries exporting specialized products and data show that countries exporting such products are characterized by high degree of diversification. So, it is not surprising that rewarding in first place number of exports and evenness of relative shares, as in our case, can lead to a substantial agreement. An exponential relation of the form $F \propto e^{\alpha S}$ is found to be quite plausible, with α assuming values close to 1.

When comparing our measure of specialization of the products Q_p with its analog computed with the Fitness algorithm, we still see a positive correlation but definitely lower ($\langle \rho_s(Q,Q^*) \rangle_t = 0.42$ on average). Following also hints in ref.[16], we therefore need to further investigate this aspect to better understand how the two specialization measures are related.

Fig. S4 is an enlarged version of Fig. 1b of the main text, which highlights the relation connecting the total export of a country with the Spearman correlation coefficient evaluated among the z_p^c of the country and the world. The palette used to color the points is representative of the entropic diversity $S_{c,20}$ evaluated in the year 2015. A vertical gradient of the colors is clearly perceivable, indicating that countries with a basket more similar to that of the world have a higher entropic diversity. A

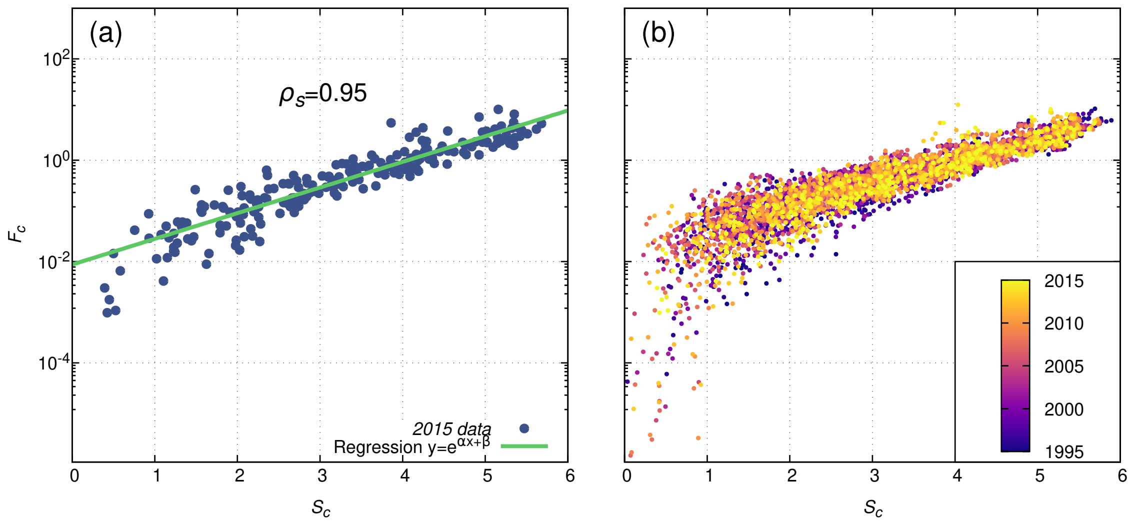

Figure S3. Panel (a): Scatter plot comparing the Fitness F and the Entropic Diversity S measures of the countries in the year 2015. A strong positive correlation is found, indicating that an exponential relation holds (green line). Panel (b): Scatter plot of panel (a) containing all the data of every year in the period 1995-2015, each colored with colors following the palette.

lighter horizontal gradient is also present, indicating that, given a fixed value of correlation, countries that have a higher total export have in general a less balanced basket.

In Fig.s S10, S11, S12 and S13 we show the 223 countries on the S-GDP$_{\text{pc}}$ plane for each year in the period between 1995-2015. As we can see, even if S_c correlates positively with GDP$_{\text{pc}}$, it is a complementary measure to it, which adds an additional non-trivial dimension that helps classifying a country's economic status. We are therefore able to distinguish countries in developed (top right corner), emerging (bottom right), underdeveloped (bottom left) and economically risky (top left).

References

1. Gaulier, G. & Zignago, S. Baci: International trade database at the product-level. the 1994-2007 version. Working Papers 2010-23, CEPII (2010).

2. UN. Commodity trade statistics database. *URL: https://comtrade.un.org* (2017).

3. WCO. Harmonic system nomenclature. *URL: http://www.wcoomd.org* (2007).

4. UNSD. Per capita gdp at current prices in us dollars. *URL: https://unstats.un.org/unsd/snaama/dnlList.asp* (2015).

5. OECD. Inflation (cpi) (indicator). *doi: 10.1787/eee82e6e-en, URL : https://data.oecd.org/price/inflation-cpi.html (Accessed on 22 Novemb. 2017)* (2017).

6. Tinbergen, J. Shaping the world economy. *Suggest. for an international economic policy', New York* (1962).

7. Glasser, D., Horn, D. & Meidan, R. Properties of certain zero column-sum matrices with applications to the optimization of chemical reactors. *J. Math. Anal. Appl.* **73**, 315 (1980).

8. Goudreé, T., Dobrinevski, A. & Bouchaud, J.-P. Explore or exploit? a generic model and an exactly solvable case. *Phys. Rev. Lett.* **112**, 050602 (2014).

9. Caraglio, M., Baldovin, F. & Stella, A. L. Export dynamics as an optimal growth problem in the network of global economy. *Sci. reports* **6** (2016).

10. Watkins, D. S. Fundamentals of matrix computations john wiley & sons. *Inc., New York, NY* (1991).

11. Roberts, A. Modify the improved euler scheme to integrate stochastic differential equations. *arXiv preprint arXiv:1210.0933* (2012).

12. Shannon, C. A mathematical theory of communication. *The Bell Syst. Tech. J.* **27**, 379 (1948).

13. Saviotti, P. P. & Frenken, K. Export variety and the economic performance of countries. *J. Evol. Econ.* **18**, 201–218 (2008). DOI 10.1007/s00191-007-0081-5.

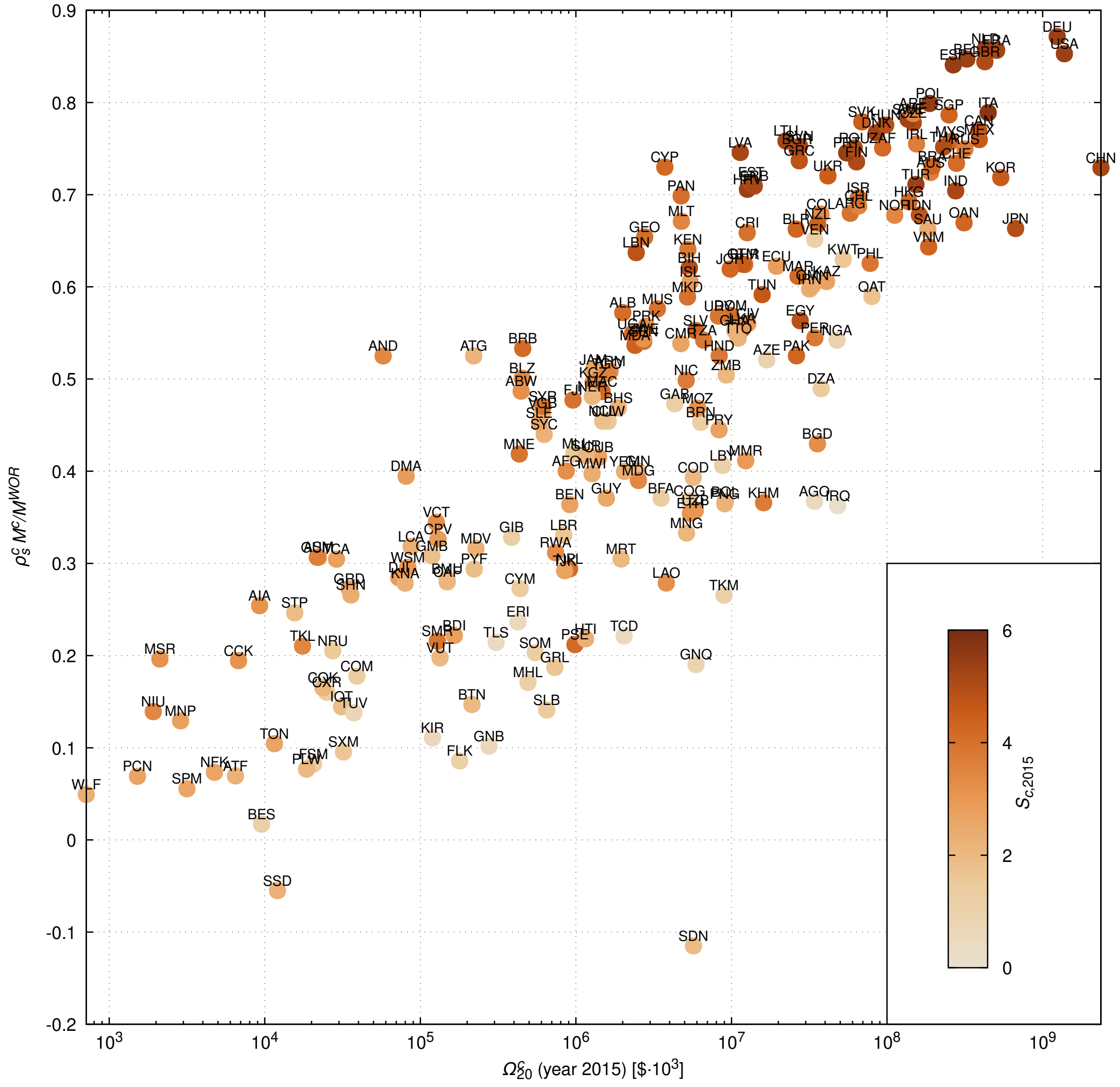

Figure S4. Total export Ω_{20}^c against the normalized Spearman's correlator $\rho_s^c M^c/M^{WOR}$ among the rankings of z_p^c of country c and the World. The color scheme is associated with the entropic diversity S_c of every country as reported in the legend.

14. Evans, L. C. & Gariepy, R. F. *Measure theory and fine properties of functions* (CRC press, 2015).

15. Tacchella, A., Cristelli, M., Caldarelli, G., Gabrielli, A. & Pietronero, L. A new metrics for countries' fitness and products' complexity. *Sci. Rep.* **2**, 723 (2012).

16. Morrison, G. *et al.* On economic complexity and the fitness of nations. *Sci. Reports* **7** (2017). DOI 10.1038/s41598-017-14603-6.

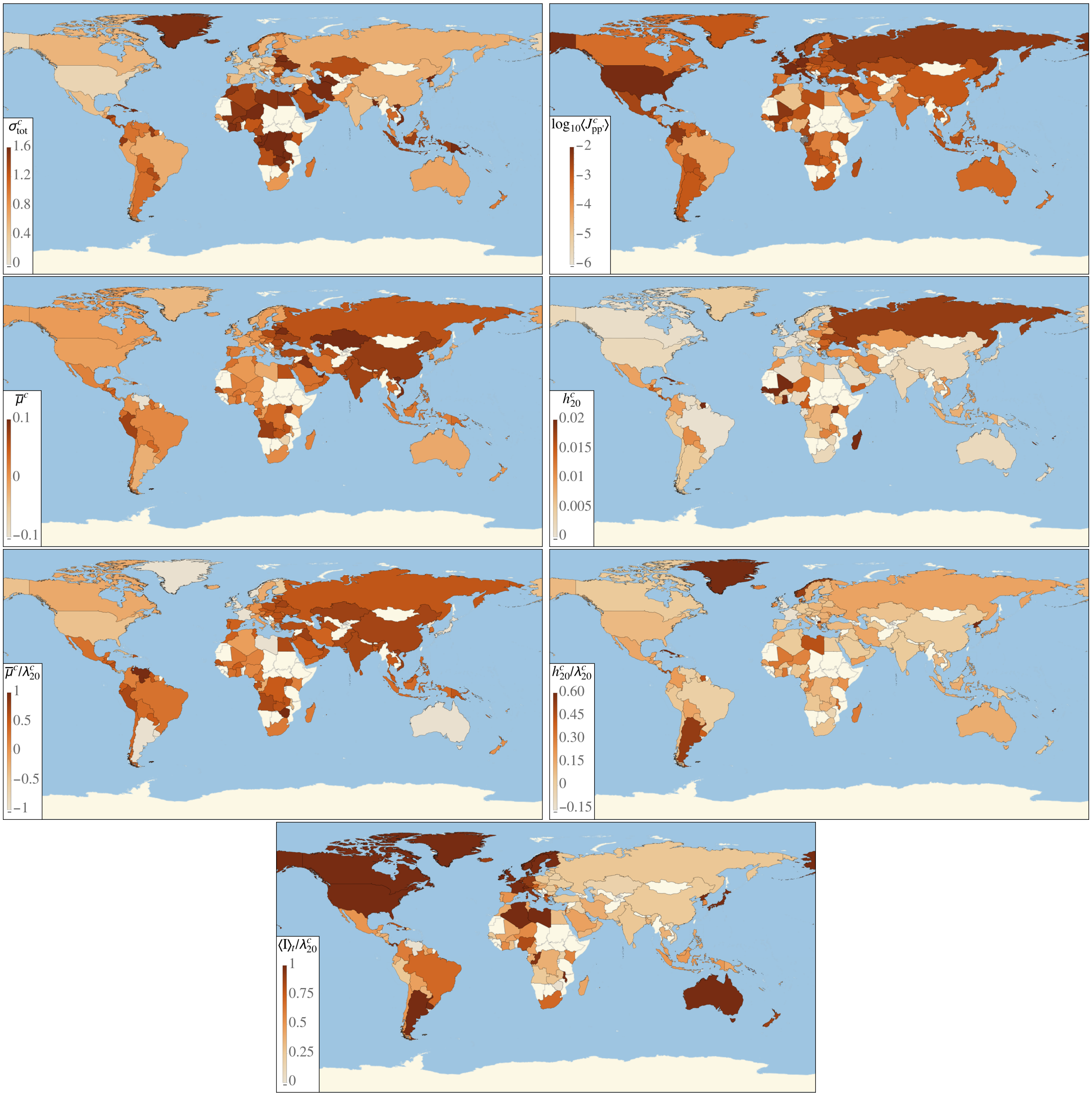

Figure S5. Parameters of the model for the 131 calibrated countries: total fluctuations a country is exposed to (σ_{tot}), the yearly average transfer rate $\langle J_{pp'} \rangle$, the average deterministic drift deprived of the inflation rate $\bar{\mu}^c$ and the growth caused by cooperativity in a country's network h^c_{20}. In the last three maps we present the contributes to the growth equation $\lambda_{20} = h_{20} + \bar{\mu} + \langle I_t \rangle_t$ normalized by the overall growth λ_{20} itself.

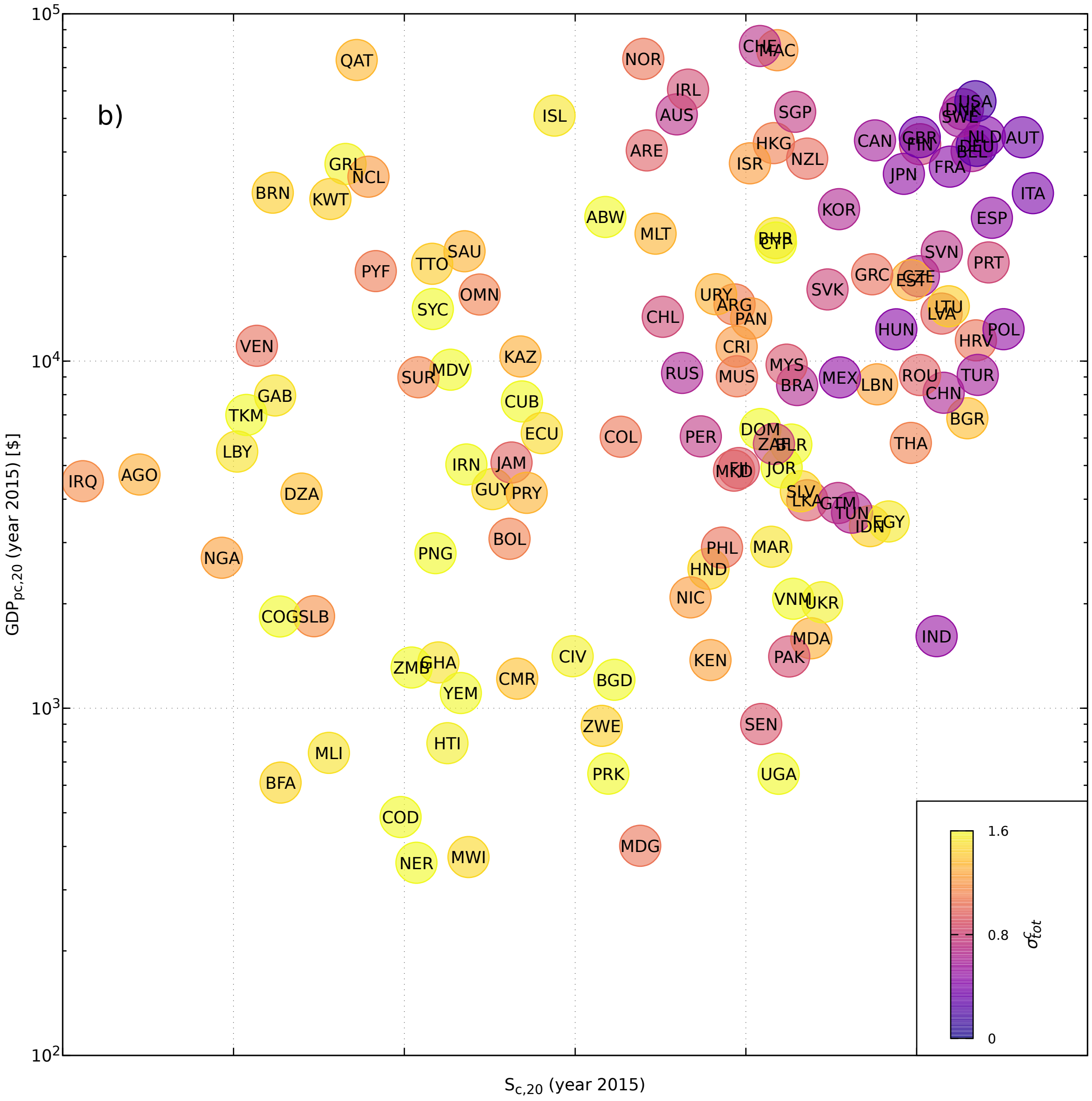

Figure S6. Enlarged figure of Fig. 4b of the main text, comprehensive of names for every country.

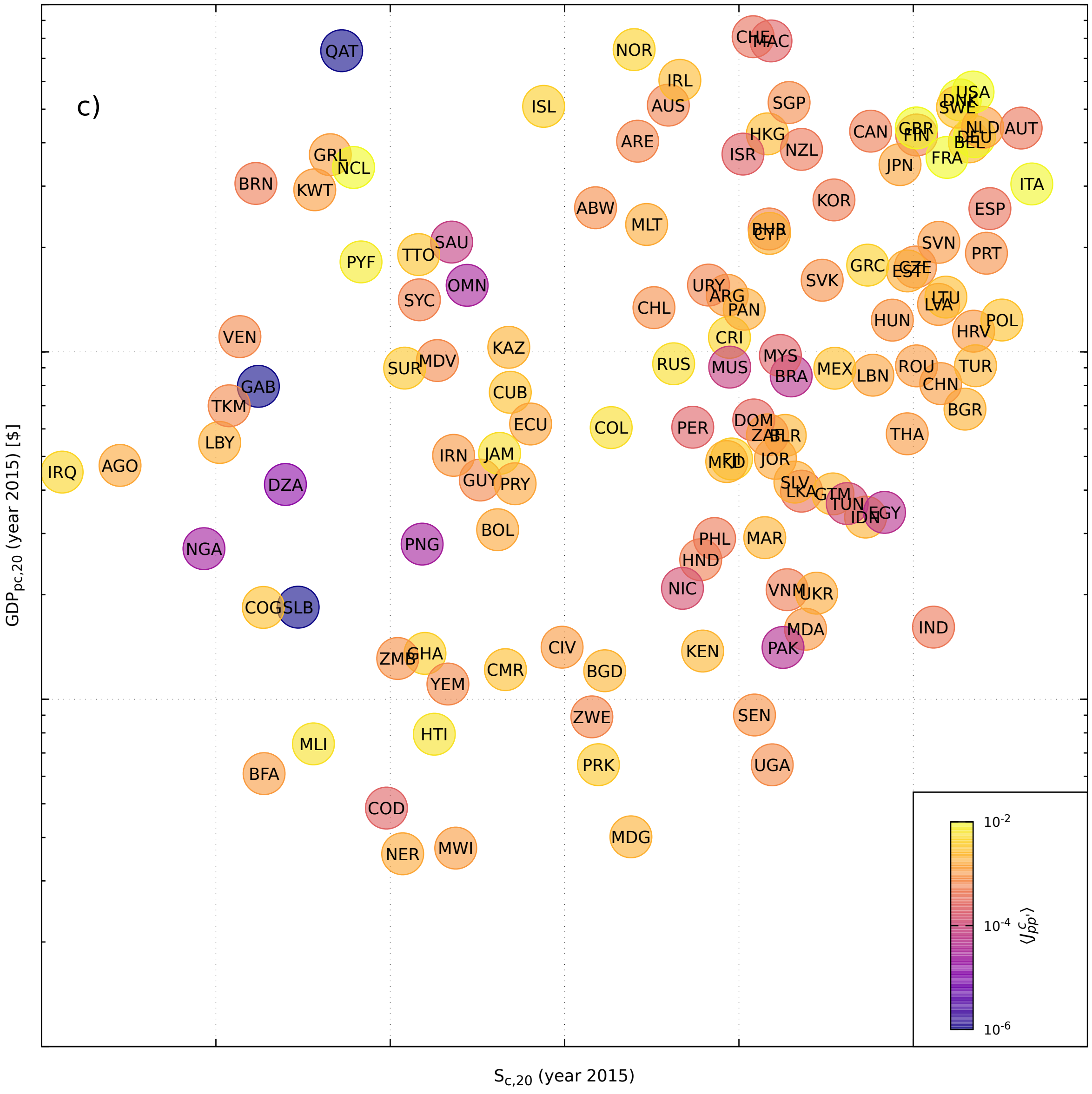

Figure S7. Enlarged figure of Fig. 4c of the main text, comprehensive of names for every country.

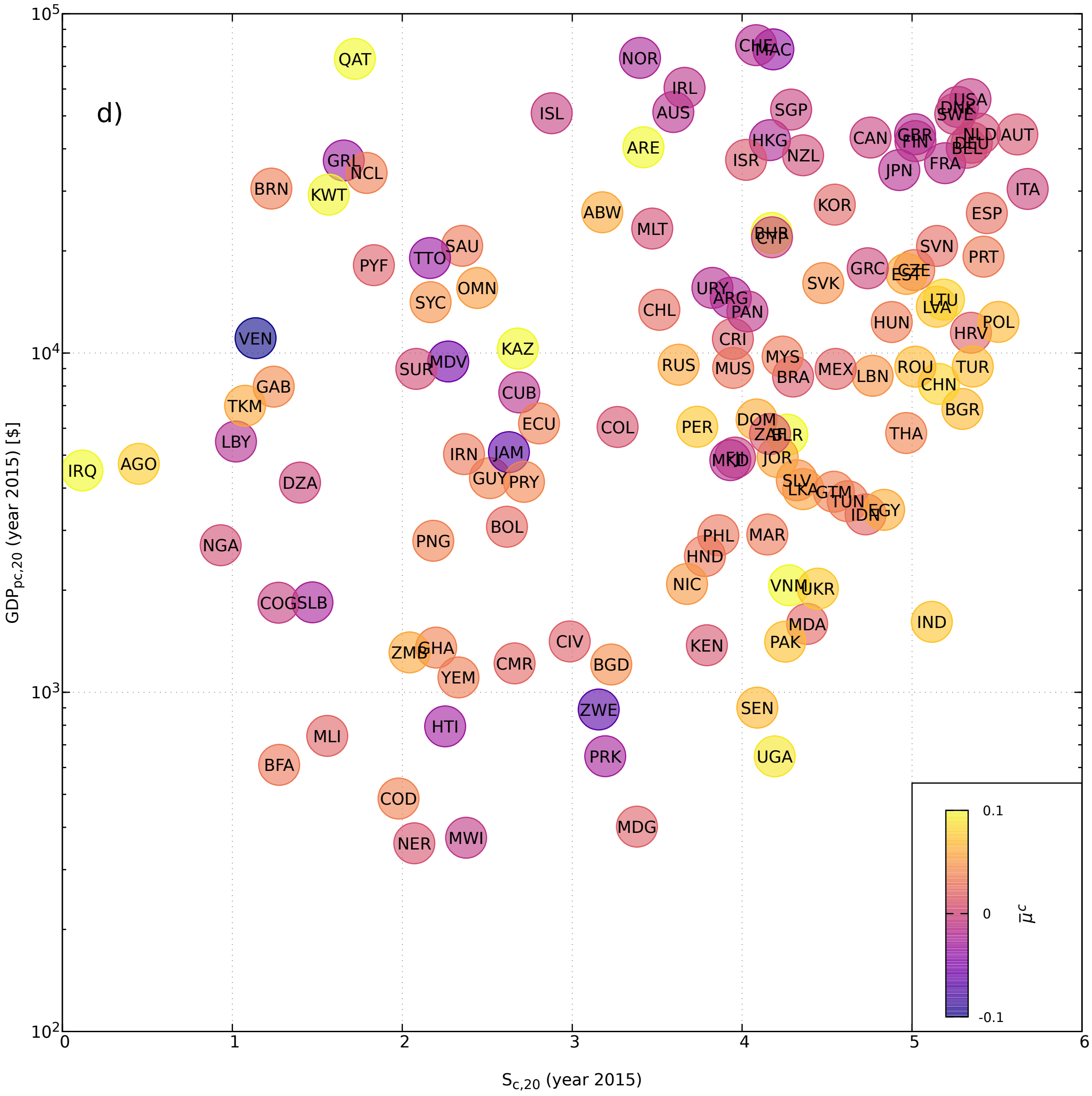

Figure S8. Enlarged figure of Fig. 4d of the main text, comprehensive of names for every country.

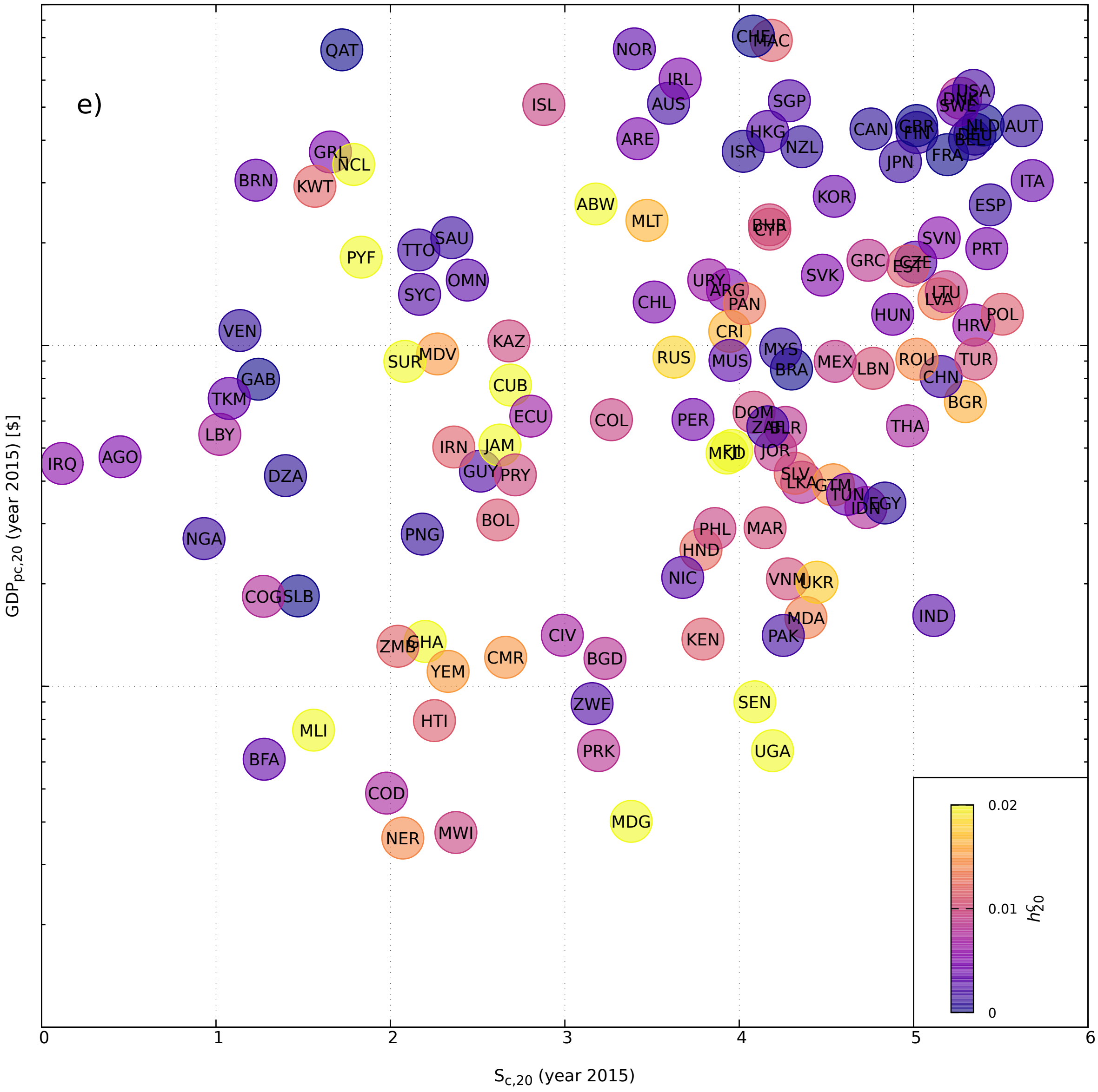

Figure S9. Enlarged figure of Fig. 4e of the main text, comprehensive of names for every country.

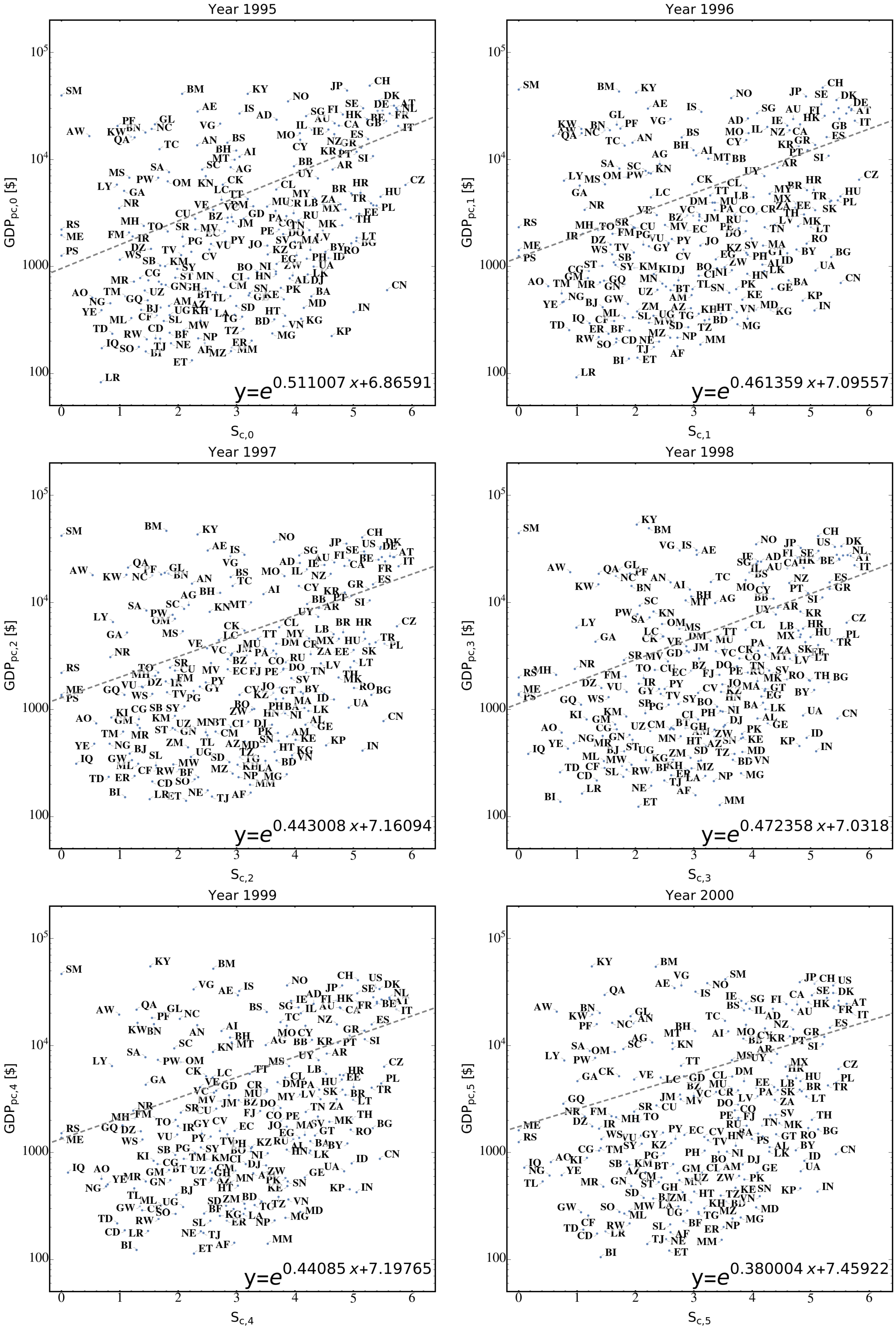

Figure S10. Countries in the S-GDP$_{pc}$ plane in the years covering the period 1995-2000. Dashed lines represent the exponential regression reported in the bottom-right corners.

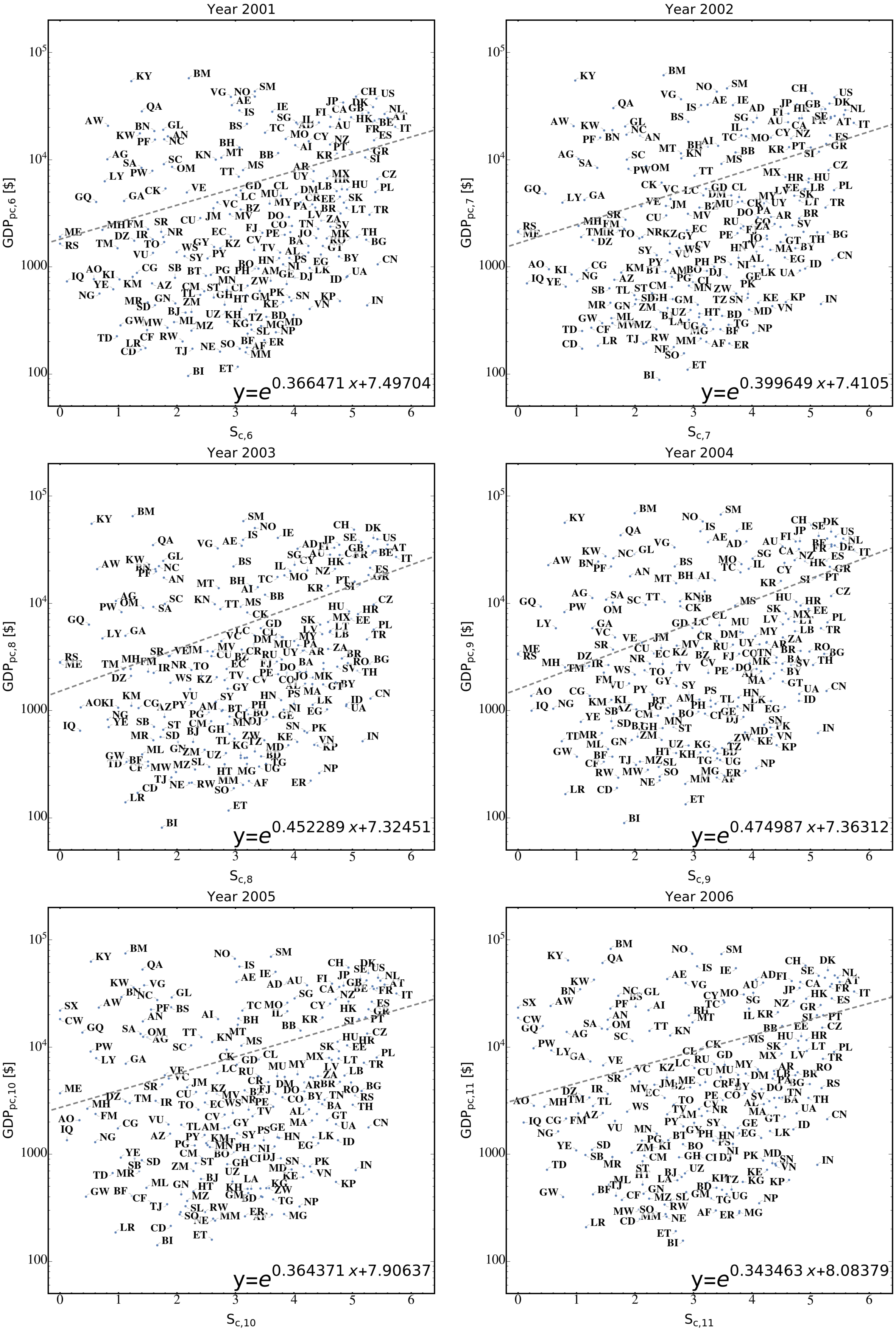

Figure S11. Countries in the S-GDP$_{pc}$ plane in the years covering the period 2001-2006. Dashed lines represent the exponential regression reported in the bottom-right corners.

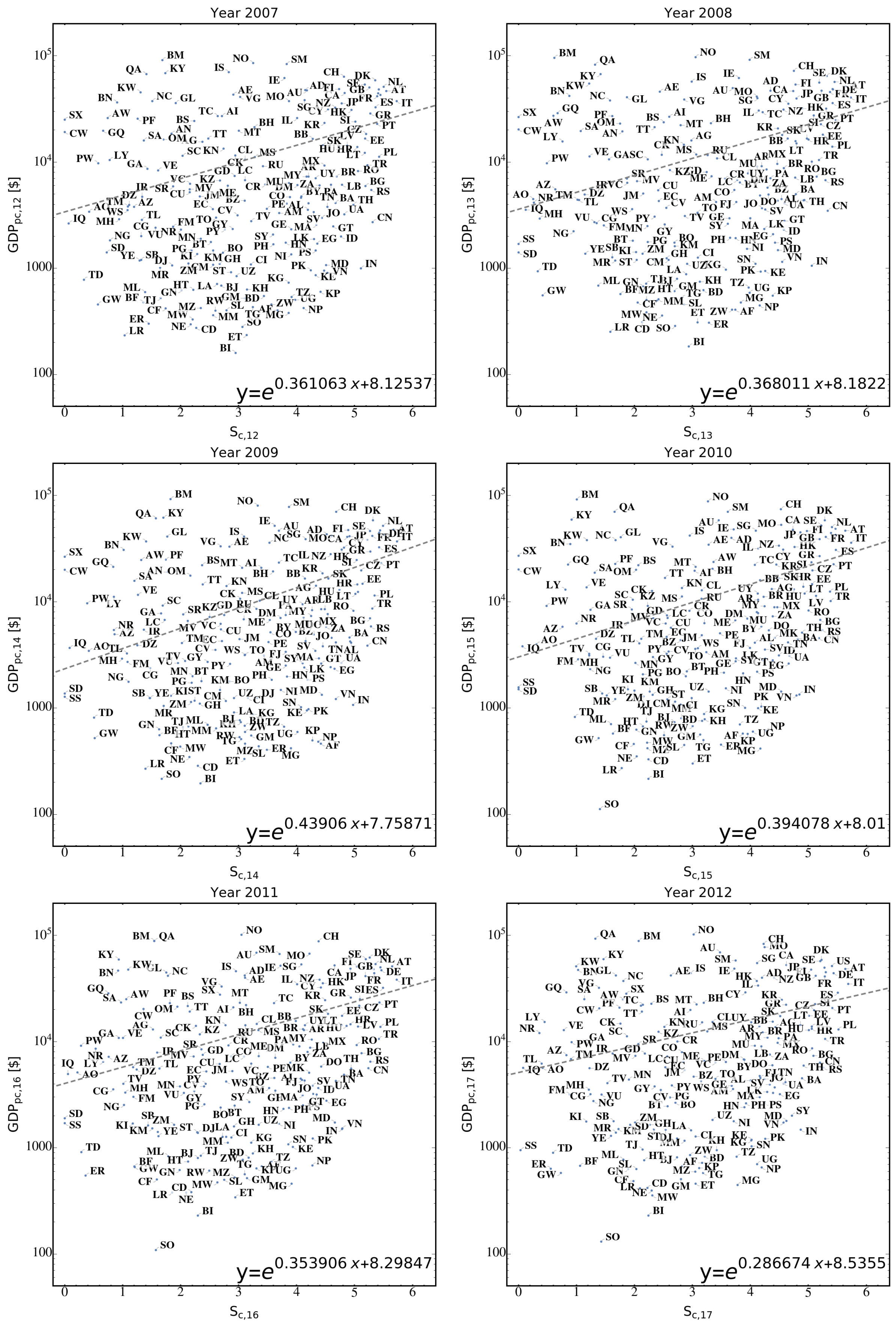

Figure S12. Countries in the S-GDP_{pc} plane in the years covering the period 2007-2012. Dashed lines represent the exponential regression reported in the bottom-right corners.

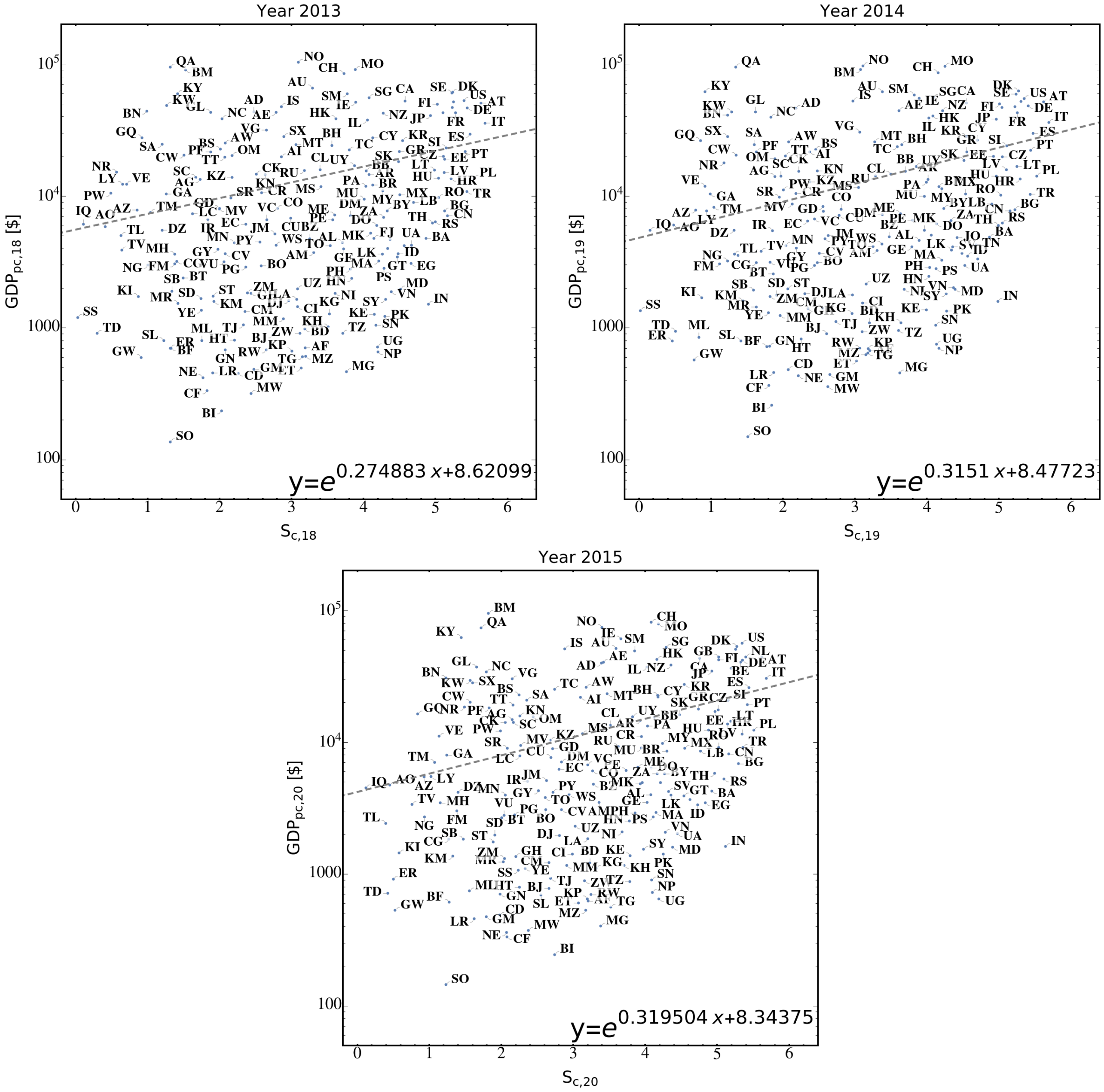

Figure S13. Countries in the S-GDP_{pc} plane in the years covering the period 2013-2015. Dashed lines represent the exponential regression reported in the bottom-right corners.
